# Brain clearance of protein aggregates in degenerative diseases: the role of microglia and astrocytes

**DOI:** 10.1186/s40035-026-00592-y

**Published:** 2026-11-04

**Authors:** Min Xiong, Yeyu Huang, Haiqiang Jiang, Zhentao Zhang

**Affiliations:** 1 Department of Neurology, Renmin Hospital of Wuhan University, Wuhan, 430060 China; 2 Innovative Institute of Chinese Medicine and Pharmacy, Shandong University of Traditional Chinese Medicine, Jinan, 250355 China; 3 State Key Laboratory of Metabolism and Regulation in Complex Organisms, College of Life Sciences, Wuhan University, Wuhan, 430000 China; 4 TaiKang Center for Life and Medical Sciences, Wuhan University, Wuhan, 430000 China; 5 Hubei Provincial Clinical Medical Research Center for Neurodegenerative Diseases, Wuhan, 430060 China

**Keywords:** Neurodegeneration, Alzheimer’s disease, Parkinson’s disease, Autophagy, Ubiquitin-proteasome system, Protein aggregation

## Abstract

Protein aggregation and impaired clearance constitute central pathological hallmarks of numerous neurodegenerative diseases, including Alzheimer’s disease, Parkinson’s disease, amyotrophic lateral sclerosis, and frontotemporal dementia. Misfolded proteins, both extracellular and intracellular, disrupt proteostasis and contribute to synaptic dysfunction, neuroinflammation, and neuronal loss. Within the central nervous system, glial cells serve as the primary mediators of pathological protein clearance. Microglia detect, phagocytose, and degrade aggregated proteins. However, under chronic pathological stress, microglial function turns from clearing pathological proteins to paradoxically facilitating their spread. Astrocytes, which provide metabolic and homeostatic support for neurons, also participate in the clearance of aggregates through phagocytosis and glymphatic drainage. Critically, crosstalk between astrocytes and microglia enhances the clearance: microglia can recruit and process aggregates internalized by astrocytes, while astrocyte-derived signals modulate the microglial phagocytic activity. Aging, genetic risk factors, or sustained neuroinflammation may reduce the efficiency of glial removal of aggregates and accelerate disease progression. Understanding the cellular and molecular underpinnings of microglial and astrocytic clearance pathways is essential for developing therapies aimed at restoring proteostasis and mitigating neurodegeneration.

## Introduction

Neurodegenerative diseases (NDs) are a group of disorders characterized by progressive loss of neurons in the central nervous system (CNS). Common NDs include Alzheimer's disease (AD), Parkinson’s disease (PD), amyotrophic lateral sclerosis (ALS), and frontotemporal dementia (FTLD). These diseases exhibit extreme clinical heterogeneity. However, they are all characterized by the deposition of pathological protein aggregates. Typically, AD is characterized by the aggregation of tau and β-amyloid (Aβ), PD by α-synuclein (α-syn) aggregation, and ALS/frontotemporal dementia (FTD) by TAR DNA‑binding protein 43 (TDP-43) and FUS proteinopathies. Most recent research has identified novel protein aggregates formed by transmembrane protein 106B (TMEM106b) [1] and TATA-binding protein-associated factor 15 (TAF15) in brains of FTLD patients [2]. Their specific roles in the progression of NDs remain largely unknown. Disease-associated mutations in these proteins increase their propensity to aggregate. Furthermore, post-translational modifications, such as truncation, phosphorylation, ubiquitination, and acetylation, drive aberrant misfolding. Once formed, the aggregated species can act as templates to induce the misfolding of their monomeric compartments. In addition, some protein aggregates can trigger the fibrillization of other proteins, a mechanism called cross-seeding (Table 1).

**Table 1:** Physiological functions and pathological features of proteins that form aggregates in neurodegenerative diseases

Proteins	Physiological function	Pathological feature	Clearance pathways	References
Tau	Microtubule-associated protein; axonal microtubule stability and assembly; facilitating the transport of substances within the axon; regulating cellular signaling and protein interactions	Multiple phosphorylation sites; truncation, acetylation, etc., promote aggregation; loss of microtubule-stabilizing function; formation of NFTs; cytotoxicity; seeding effect	Recognition receptors: LRP1, HSPG, FcγR. Soluble tau: UPS. Aggregated tau and PHFs/NFTs: autophagy pathways and glymphatic/lymphatic clearance	[3–5]
Aβ	Produced by proteolytic cleavage of APP; inhibiting microbial infections; regulating synaptic plasticity; promoting recovery of brain tissue damage; repairing the blood‒brain barrier	Insoluble cross-β structure; α-sheet-structure-formed aggregation core; hydrophobic segments and phosphorylation; formation of amyloid plaques; cytotoxicity; seeding effect; exacerbating tauopathy.​	Recognition receptors: TREM2, LRP1, CD36, TLR2/4, RAGE, FcγR. Intracellular Aβ: UPS and lysosome; Extracellular Aβ: glymphatic/lymphatic and LRP1-mediated BBB transforming clearance	[6–8]
α-Syn	Abundant in the presynaptic terminals of neurons; influencing the dynamics of the SNARE complex; regulating synaptic vesicles and neurotransmitters; cellular signal interaction; may participate in gene regulation and neuro-immune responses	Intrinsically disordered; membrane binding and lipid-modifiable; aberrant gene mutant and dose-response; loss of synaptic transmission function; Lewy bodies and pathological propagation; cytotoxicity	Recognition receptors: TLR2/4, CD36, LAG3, LRP1. Soluble α-syn: CMA and non-specific UPS. Aggregated α-syn: autophagy and lysosome	[9–11]
TDP-43	DNA/RNA binding protein; pre-mRNA splicing; regulating mRNA stability and extracellular transport localization; DNA binding and regulating of gene expression	Prion-like low-complexity domain; intrinsically disordered; loss of nuclear function; pathological propagation; cytotoxicity; seeding effect	No clear extracellular receptor. Soluble TDP-43: UPS. Aggregated TDP-43: autophagy	[12–14]
TMEM106B	A type II transmembrane protein located on the membranes of late endosomes or lysosomes; lysosome movement and transport; lysosome pH regulation; maintaining the stability of neuronal dendrites; generating myelin sheaths	The C-terminal fragment (120–254) is rich in β-strands, which is prone to aggregation; forms aggregates in neurodegenerative diseases and aged brain	Not clear	[1, 15, 16]
TAF-15	DNA/RNA binding protein belonging to FET family; pre-mRNA splicing; mRNA transport; DNA binding	Prion-like low-complexity domain; intrinsically disordered; mechanism still under investigation	Not clear	[2, 17, 18]

Aβ, amyloid-beta; α-Syn, alpha-synuclein; TDP-43, TAR DNA-binding protein (43 kDa); TMEM106b, transmembrane protein 106B; TAF-15, TATA-box binding protein associated factor 15; PHFs, paired helical filaments; NFTs, neurofibrillary tangles; APP, amyloid precursor protein; SNARE, soluble N-ethylmaleimide-sensitive factor attachment protein receptors; CMA, chaperone-mediated autophagy; UPS, ubiquitin-proteasome system

The pathological protein aggregates are insoluble, protease-resistant, and refractory to clearance. Converging lines of evidence indicate that the protein aggregates are not only pathological markers, but also play key roles in neuronal dysfunction and degeneration [3, 4, 6, 8, 10]. Therefore, understanding how the brain clears or processes protein aggregates is crucial for elucidating disease mechanisms and developing therapeutic strategies. In this review, we summarize existing data on the molecular mechanisms underlying the formation and the clearance of pathological protein aggregates, with a focus on the roles of microglia and astrocytes.

## Literature search strategy

A systematic literature search was performed to identify peer-reviewed articles on glial-mediated clearance of pathological protein aggregates in NDs. The search was conducted in PubMed/MEDLINE, Web of Science Core Collection, and Google Scholar from database inception to 2026. The search strategy combined controlled vocabulary and free-text keywords across three domains: (1) neurodegenerative diseases (“Alzheimer’s disease”, “Parkinson’s disease”, “Amyotrophic lateral sclerosis”, “Frontotemporal dementia”); (2) pathological protein aggregates (“amyloid-beta”, “tau”, “alpha-synuclein”, “TDP-43”, “FUS”, “protein aggregation”, “misfolded proteins”); (3) clearance mechanisms (“microglia”, “astrocytes”, “phagocytosis”, “autophagy”, “ubiquitin–proteasome system”, “chaperone-mediated autophagy”, “glymphatic system”, “meningeal lymphatic”, “TREM2”, “LRP1”, “complement receptors”, “Fc receptors”, “Toll-like receptors”, “NLRP3 inflammasome”, “cGAS-STING”); and (4) therapeutic strategies targeting α-syn、Aβ and tau or therapeutic strategies in ALS and FTD. Boolean operators (AND, OR) were used to combine terms. Inclusion criteria were: (1) original research articles, clinical trials, and systematic reviews; (2) English language; (3) studies reporting on molecular or cellular mechanisms of clearance of Aβ, tau, α-syn, TDP-43, or FUS by microglia or astrocytes, including UPS, autophagy, phagocytosis, and glymphatic/lymphatic pathway; and (4) studies using human subjects, human-derived tissues/cells, or animal models of NDs. Exclusion criteria were: (1) non-English articles; (2) conference abstracts, editorials, commentaries, or opinion pieces; (3) studies focused exclusively on non-neurological diseases or non-protein aggregate pathologies.

While every effort was made to comprehensively capture the literature through the described search strategy, the authors acknowledge that the selection of studies may be subject to publication bias (positive results more likely to be published) and language bias (English-only inclusion). The final synthesis prioritized studies published within the last 10 years, while seminal older works were included where historically relevant. Furthermore, the rapid expansion of the field means that some very recent findings may not be included.

## Clearance of pathological protein aggregates: cellular and extracellular systems

The production and clearance of proteins are tightly regulated in the brain. In response to multiple stresses, including endoplasmic reticulum (ER) stress, oxidative insults, heat shock, hypoxia, and accumulation of misfolded or aggregated proteins, cells may adopt a series of strategies to respond to these threats. Clearance of pathological protein aggregates (PPAs) should be studied at both macroscopic and microscopic scales. Macroscopically, proteins can be cleared either intracerebrally or peripherally. Microscopically, these proteins can be cleared either by the intracellular system or by the extracellular system.

Most PPAs in NDs are primarily formed in the brain parenchyma, mostly in neurons. Intriguingly, α-syn is also present in the gut and other peripheral tissues and supports pathological propagation through the “gut-brain axis”. However, primary accumulation in the brain still originates from neuronal expression.​​ The clearance of PPAs by microglia and astrocytes forms a highly coordinated multicellular network critical for CNS homeostasis. Dysfunction of this network represents a core pathogenic event across all major NDs. The network is activated when glial cells detect neurodegeneration-associated molecular patterns (NAMPs) and "eat-me" signals exposed on compromised or dying neurons. Microglia act as first responders, rapidly engulfing large protein aggregates via recognition receptors. Astrocytes complement this function by efficiently clearing smaller aggregates and synaptic elements, albeit with lower overall capacity for large inclusions.

Engulfed material is eliminated through a cascade of intracellular degradation pathways. Both glial types primarily rely on the lysosomal system, where cathepsins and other acid hydrolases mediate aggregate breakdown. The ubiquitin–proteasome system (UPS) additionally degrades soluble, ubiquitinated monomers and oligomers. Autophagy, particularly chaperone-mediated autophagy (CMA), also serves as a critical complementary mechanism for intracellular aggregate clearance.

A defining feature of this network is the intercellular material transfer. Under excessive proteotoxic stress, partially degraded protein fragments or undegraded aggregates are released via tunneling nanotubes (TNTs) or extracellular vesicles (EVs) and captured by neighboring glial cells, which then initiate their own full clearance program.

This tightly regulated intercellular crosstalk underpins the dual nature of glial activation in NDs. Early-stage activation is predominantly neuroprotective, promoting efficient protein clearance, tissue repair, and neuronal survival. However, chronic exposure to pathological proteins drives a maladaptive phenotypic shift to a proinflammatory, neurotoxic state characterized by downregulation of phagocytic receptors, impaired lysosomal and autophagic function, and excessive release of proinflammatory cytokines and reactive oxygen species (ROS). This creates a self-perpetuating vicious cycle, in which glial dysfunction exacerbates protein aggregation, which in turn further impairs glial function, driving progressive neurodegeneration. In the following, we will discuss aspects of PPA targeting, recognition and phagocytosis of glial cells, intracellular degradation, delivery of substances between cells, and waste removal via the lymphatic system.

### Upstream tagging system governing glial-mediated protein clearance

Regarding how targets in the CNS are defined that need to be eliminated, recent studies have proposed a concept that the clearance of abnormal proteins by glial cells is not random ingestion, but is precisely controlled by an “upstream tagging system” [19–21]. That is, before pathological proteins are cleared, they need to be modified by specific "tagging signals" so that they can be recognized, encapsulated, and enter the degradation pathway by microglia and astrocytes.

Diverse signals, such as phosphatidylserine (PS) externalization, immune opsonins, lipoprotein modifications, aberrant glycosylation, the complement system, and defined “don’t-eat-me” signals, collectively form a “glial recognition code” that determines whether glial cells initiate phagocytosis, inflammatory amplification, or synaptic pruning during neurodegeneration.

Among these mechanisms, PS externalization represents the most classical “eat-me” signal. Under physiological conditions, PS is confined to the inner leaflet of the plasma membrane. However, neuronal stress, synaptic dysfunction, mitochondrial impairment, and membrane perturbation induced by tau, Aβ, and α-syn aggregates promote abnormal PS exposure on degenerating neurons and dysfunctional synapses. The exposed PS is subsequently recognized by microglial receptors, thereby initiating phagocytic clearance [19, 22].

Lipoprotein modifications constitute another essential upstream tagging system. In AD pathology, APOE not only functions as a lipid transporter, but is also associated with tau and Aβ aggregates, acting as an opsonin-like bridge that facilitates uptake of pathological substrates by triggering receptor expressed on myeloid cells 2 (TREM2)-positive microglia. Increasing evidence further suggests that the APOE–TREM2 axis represents a major pathway coordinating astrocyte–microglia communication, lipid redistribution, and stabilization of the disease-associated microglia (DAM) transcriptional program during neurodegeneration [23]. Disease-associated variants such as *TREM2* R47H and *APOE4* disrupt this process, leading to impaired clearance.

In addition, aberrant glycosylation may function as a recognition signal for microglia and astrocytes via distinct molecular mechanisms in neurodegenerative disorders. For microglia, abnormal glycosylation of core membrane receptors directly alters their function. For example, defective glycosylation of TREM2 weakens its ligand-binding and signaling, impairing microglial phagocytosis of pathological proteins like Aβ. Hyper-glycosylation of CD33 activates its immunoreceptor tyrosine-based inhibitory motif (ITIM) domains to further suppress phagocytosis. Aberrant glycosylation of receptor for advanced glycation end products (RAGE) increases its affinity for pro-inflammatory ligands, activating downstream pathways to drive microglia toward the pro-inflammatory phenotype [24].

The complement system constitutes one of the most prominent upstream tagging mechanisms in NDs. During early disease stages, the classical complement pathways become aberrantly activated and preferentially deposit on functionally impaired but still viable synapses, leading to synaptic opsonization. Microglia subsequently recognize these tagged synapses through complement receptors, triggering excessive synaptic engulfment and pathological synaptic pruning. Nonetheless, the diversity of complement has now become a new research direction in the field of NDs. Complement receptor 1 (*CR1*) is a core risk gene for late-onset AD identified by genome-wide association studies (GWAS). In 2012, Hazrati et al. first demonstrated that the copy number variation of the 18-kilobase low-copy repeat 1 (LCR1) in the *CR1* gene is the true causal variant underlying the GWAS signals. The risk isoform CR1-S (CR1-2), which carries two copies of LCR1, increases the risk of AD by 1.84-fold. Notably, the protein expression level of this risk isoform in human brain tissue is significantly lower than that of the non-risk isoform CR1-F (CR1-1). Additionally, the two isoforms demonstrate distinct subcellular localization patterns. The CR1-F is localized to the ER–Golgi intermediate compartment, while the CR1-S accumulates in lysosomes, indicating differences in their protein processing and degradation pathways [25]. In 2025, Daskoulidou et al. further resolved the paradox of “lower expression but higher risk” using human induced pluripotent stem cell (iPSC)-derived microglia and astrocytes. They confirmed that although CR1-S expression is 5- to 8-fold lower, its additional C3b/C4b-binding site confers higher catalytic efficiency per molecule for the conversion of C3b to iC3b. The locally generated iC3b is sufficient to offset the reduction in expression level, ultimately significantly enhancing the phagocytic capacity of glial cells. This enhanced phagocytosis is a double-edged sword: while it accelerates Aβ clearance, leading to lower brain Aβ burden in CR1-S carriers, it simultaneously causes excessive synaptic loss and neuroinflammation, which constitutes the core mechanism underlying the elevated AD risk associated with this variant [26]. Collectively, these studies systematically elucidate the molecular basis by which CR1 diversity influences AD susceptibility and disease progression through regulating the balance of complement activation and glial phagocytic function. These findings not only refine the immune pathogenic model of AD, but also provide a critical theoretical foundation for the development of *CR1* genotype-based individualized precision therapeutic strategies.

In contrast to “eat-me” signals, the CNS also possesses critical “don’t-eat-me” checkpoint systems that normally restrain excessive glial phagocytosis. The “don’t eat me” signals are a class of cell surface inhibitory molecules that maintain CNS homeostasis by preventing excessive phagocytosis of healthy neurons and functional synapses. Dysregulation of these signals in NDs leads to aberrant clearance of neural elements, representing a critical mechanism underlying synaptic loss and cognitive decline. The most well-characterized pathway is the CD47–signal regulatory protein α (SIRPα) axis. CD47 is widely expressed on neurons and synapses, and binds to SIRPα on microglia, which recruits phosphatases via intracellular ITIM motifs to suppress phagocytic signaling. In AD, downregulation of microglial SIRPα expression impairs this protective checkpoint, driving complement-independent excessive synaptic pruning even in the absence of Aβ plaques. Complement system regulators act as complement-specific "don't eat me" signals. SRPX2 (Sushi repeat-containing protein X-linked 2) inhibits the C1q-mediated classical pathway activation [27]. CSMD1 (CUB and Sushi Multiple Domains 1) acts as a CNS-specific inhibitor of C3 convertase [20]. CD55 accelerates the decay of C3/C5 convertases to decrease the production of downstream complement intermediates, while CD59 directly blocks the assembly of the membrane attack complex [21, 28]. CR1 not only functions as an opsonin receptor, but also negatively regulates complement activation. The isoform diversity of CR1 directly modulates the balance between complement inhibition and phagocytosis (as discussed above). The sialic acid-siglec pathway represents another critical checkpoint: sialic acid residues on neuronal glycoproteins bind to siglec family receptors on microglia to deliver inhibitory signals [29]. In neurodegenerative conditions, the balance between “don’t eat me” and “eat me” signals is profoundly disrupted. Normalizing glial phagocytic activity by targeting these inhibitory checkpoints or inhibiting those “eat-me” signals, has emerged as a promising therapeutic strategy to mitigate neurodegeneration.

Collectively, current evidence supports the concept that the glial-mediated clearance in NDs is not driven by pathological protein accumulation alone, but rather orchestrated by multiple interconnected upstream tagging systems. These integrated signaling networks ultimately determine whether glial responses remain protective or transition toward chronic neuroinflammation and maladaptive phagocytosis, thereby representing promising therapeutic targets for neurodegenerative disorders.

### Microglia: recognition, phagocytosis, and immunomodulation

Pathological proteins within cells can be transferred to the extracellular space through cellular penetration during cell damage, apoptosis, or death, or through EV release, and subsequently propagate to neighboring cells. When the burden of pathological proteins in the extracellular environment increases, microglia and astrocytes are responsible for maintaining homeostasis [30].

Microglia are the principal innate immune effector cells of the CNS and execute multiple functions that can be organized conceptually into three tightly linked aspects: target recognition, phagocytosis, and context-dependent immunomodulation. The active state of microglia was first observed in 1987, directly linking the specific microglial state to AD patients [31]. Recent single-cell and spatial transcriptomic studies have refined this framework by demonstrating that these functions are mediated by complex microglial states rather than a single, uniform “activated” phenotype. Disease and aging initiate heterogeneous activation programs, connecting membrane receptors to the intracellular metabolic progress, thereby determining whether microglia act protectively or pathologically [32–35].

Since the introduction of DAM concept in NDs, the role of microglia in disease progression has become more clear. DAMs represent an activated subpopulation of microglia that respond to pathological protein deposition or tissue damage within the CNS. The transcriptional profile of DAMs is markedly distinct from that of homeostatic microglia [36–38]. In the homeostatic but ‘alert’ state, microglia maintain high expression of some homeostatic markers, such as TMEM119 (transmembrane protein 119), P2RY12 (purine receptor P2Y12), and CX3CR1 (CX3C motif chemokine receptor 1), which provide nutritional support for neurons and regulate synaptic plasticity [39–41]. The formation of DAMs typically begins when microglia sense NAMPs, which often include PPAs such as Aβ, tau, or α-syn. These NAMPs are recognized through multiple receptors, recognizing “eat-me” signals (phosphatidylserine, calreticulin, etc.) via membrane receptors, subsequently phagocytosing and degrading them. These “eat-me” signals are usually located at the cellular membrane, cellular debris, or extracellular compartment. Microglia employ a diverse array of membrane receptors to recognize and bind PPAs, which can be broadly categorized into two functional classes: receptors that directly mediate tau/Aβ recognition and internalization, and receptors that primarily exert immunomodulatory effects.

The first class, responsible for the direct uptake of extracellular pathological proteins (including monomers and aggregates/fibrils), primarily includes heparan sulfate proteoglycans (HSPGs), low-density lipoprotein receptor-related protein 1 (LRP1), and Fc gamma receptors (FcγRs). HSPGs expressed on the microglial cell surface serve as initial docking sites for extracellular pathological proteins. The heparan sulfate side chains of HSPGs carry abundant negatively charged binding sites, which can engage pathological proteins such as tau and Aβ. Binding of tau or Aβ to membrane HSPGs triggers widespread membrane ruffling and drives the formation of macropinosomes, which engulf the bound proteins along with the surrounding extracellular fluid into the cell. Furthermore, when macropinosomes cluster within cholesterol-rich lipid raft microdomains, they initiate lipid raft-dependent, actin-based macropinocytosis to mediate non-selective bulk uptake. In addition, pathological proteins can enter cells through two receptor-mediated endocytic routes. First, these proteins can directly bind to LRP1 to form receptor-ligand complexes, which are internalized via clathrin-mediated endocytosis. Second, pathological proteins may first assemble into antigen–antibody complexes, signaling through FcγRs to promote cellular entry, also via the clathrin-mediated pathway [42–44].

The second class of receptors, such as TREM2, Tyro3-Axl-MERTK Receptor Tyrosine Kinase Family (TAMs), CD33, and partial pattern recognition receptors (PRRs, e.g., TLR2/4, nucleotide-binding oligomerization domain-like receptors [NLRs], and cGAS), mainly performs immune regulatory functions. These receptors do not directly capture tau molecules, but they can reconfigure the state of microglia (such as the DAM phenotype) and influence inflammatory responses, phagocytic potential and even nerve damage through signaling pathways. Based on this mechanism, microglia may trigger inflammation and even neuronal damage while clearing pathological proteins. TREM2 is highly expressed by microglia, serving as a critical checkpoint in DAM transcriptional programming. Deficiency of TREM2 impairs microglial clustering around Aβ plaques, exacerbating neuronal damage. As a surface receptor on microglia, TREM2 initiates signaling pathways by interacting with the adaptor protein TYROBP (TYRO protein tyrosine kinase binding protein, also known as DAP12), thereby promoting phagocytic activation and survival of microglia. The surface level of TREM2 can be regulated by its shedding (generation of soluble sTREM2 via ADAM10 cleavage) or transport from intracellular TREM2 reservoirs. Upon ligand binding, SRC family tyrosine protein kinases phosphorylate the tyrosine residues within the immunoreceptor tyrosine-based activation motif (ITAM) domain of DAP12. This subsequently recruits spleen tyrosine kinase (SYK), initiating downstream activation of protein kinase B (AKT) and extracellular signal-regulated kinase (ERK) pathways, which together converge on the mechanistic target of rapamycin (mTOR) signaling axis. AKT suppresses GSK3β activity, thereby reducing tau hyperphosphorylation while also enhancing mTOR expression to support microglial proliferation and survival. In parallel, ERK promotes anti-apoptotic signaling and regulates protein synthesis, ultimately influencing the proliferation, differentiation, and clearing-associated function of microglia [23]. Meanwhile, the TREM2-DAP12 signaling pathway suppresses the production of proinflammatory cytokines following TLR activation [45]. The TAM receptors (Tyro3, Axl, and MERTK) are a family of evolutionarily conserved receptor tyrosine kinases. Similar to the TREM2 pathway, TAM activation also initiates the recruitment of SYK. The TAM receptor family is activated by two primary ligands: growth arrest-specific 6 (GAS6) and protein S (PROS1) [46–48]. While GAS6 exhibits high affinity for all three TAM receptors, with strongest binding to Axl, PROS1 preferentially binds to and activates Tyro3 and MERTK [47]. However, when the inhibitory receptor CD33 recognizes sialoglycans, it recruits inhibitory phosphatases such as SHP (Src homology region 2 domain-containing phosphatase), which antagonizes phagocytic signaling mediated by SYK. This suppresses microglial activation and diminishes their capacity for clearance. PRRs such as TLR4, TLR2, and RAGE, not only directly recognize tau, but also sense NAMPs and activate the classical NF-κB signaling axis, driving microglia to release large amounts of pro-inflammatory cytokines, triggering a cytokine storm [49, 50]. NLRs represent a major superfamily of PRRs in glial cells. In the CNS, they are primarily classified into two major subfamilies based on their N-terminal effector domains: the NLRP subfamily, which contains a pyrin domain, and the NLRC subfamily, which contains a caspase activation and recruitment domain (CARD) [51]. Among these, NLR Family Pyrin Domain Containing 3 (NLRP3) is a critical receptor that mediates microglial activation and phenotypic transition. Its expression is primarily regulated by the transcription factor NF-κB. Consequently, any process that activates NF-κB signaling, such as stimulation of TLRs or NOD1/2, can upregulate NLRP3 expression. Notably, NLRP3 does not recognize specific molecular structures but rather senses general intracellular "danger states" induced by diverse stimuli [52]. Upon activation, NLRP3 first oligomerizes to form a platform that recruits the adaptor protein ASC (apoptosis-associated speck-like protein containing a CARD), which then polymerizes into large speck structures. The CARD domain of ASC subsequently recruits pro-caspase-1, bringing it into close proximity to undergo autocatalytic cleavage and form active caspase-1 dimers. Active caspase-1 then cleaves pro-inflammatory cytokine precursors, leading to the release of mature, biologically active cytokines. Accumulating evidence indicates that chronic NLRP3 stimulation impairs microglial phagocytic capacity while paradoxically enhancing the expression and release of pro-inflammatory cytokines [53, 54]. The cyclic GMP-AMP synthase–stimulator of interferon genes (cGAS-STING) pathway is another key intracellular innate immune pathway alongside the NLR family that plays a central role in NDs. It is highly expressed primarily in microglia and astrocytes and initiates immune responses by recognizing aberrantly localized cytoplasmic double-stranded DNA. In neurodegenerative conditions, PPAs can cause massive leakage of mitochondrial DNA (mtDNA) and genomic DNA into the cytoplasm by directly damaging the mitochondrial respiratory chain, inhibiting mitophagy and disrupting nuclear envelope integrity. These abnormal DNA molecules are recognized by cGAS, which catalyzes the synthesis of the second messenger cGAMP. This in turn activates the endoplasmic reticulum membrane protein STING, inducing its conformational change and translocation to the Golgi apparatus, where it recruits and phosphorylates the TANK-binding kinase 1** (**TBK1). Activated TBK1 simultaneously triggers the NF-κB downstream pathways, leading to robust expression of INF-I and pro-inflammatory cytokines. Importantly, the cGAS-STING pathway also activates the NLRP3 inflammasome, forming a potent cGAS-STING-NLRP3 inflammatory amplification axis that mediates microglial pyroptosis and the release of pathological protein seeds, thereby promoting the spread of pathology throughout the brain [55].

There are also receptors sharing characteristics of both classes of receptors mentioned above. Lymphocyte activation gene-3 (LAG3) is a transmembrane protein and an immune checkpoint receptor that belongs to the immunoglobulin superfamily. On the one hand, the expression of LAG3 is upregulated when microglia sense excessive pro-inflammatory factors. When the expression of LAG3 is reduced or inhibited, microglia will produce more inflammatory mediators, such as nitric oxide (NO), in response to IFN-γ stimulation, indicating that LAG3 plays a role in inhibiting inflammatory activation [56]. Intriguingly, LAG3 has been proven to bind misfolded preformed fibrils (PFFs) of α-syn, foster the endocytosis and spread of α-syn pathology, and even upregulate the toxicity of PFFs [57]. However, this phenomenon has not been observed in other PFFs or physical proteins, requiring stronger evidence. Scavenger receptors are a special type of PRR. They can not only recognize antigen–antibody complexes for endocytosis, but also regulate immune reactions [58, 59]. Complement receptors (CRs, e.g., CR3, CR4) are mainly expressed on microglia membranes. The formation of the antigen–antibody complex induces a conformational change in the Fc part of the antibody, which then forms a complex with complement. Finally, the complement complex will be recognized through CR3/4 and phagocytosed by microglia. Researchers have indicated that CR4 can selectively bind to fibrillar tau and that CR3 can selectively bind to fibrillar Aβ [60, 61]. CR1 not only mediates phagocytosis but also accelerates the degradation of C3 convertases to inhibit complement cascade amplification, thereby exerting potent immunomodulatory effects [62].

These immune regulatory signals do not operate independently. TREM2/DAP12-SYK not only promotes phagocytic and DAM phenotypic metabolic reprogramming, but can also cross-activate NF-κB to promote inflammation. TLR/NLRP3 induces a strong inflammatory state, and through feedback from inflammasome products, it promotes further transformation of the microglial phenotype toward a pro-inflammatory profile. The cGAS-STING pathway engages in a bidirectional negative feedback regulation with TREM2. TREM2 inhibits cGAS-STING activation by promoting mitophagy to clear damaged mitochondria and reduce mtDNA release. Conversely, chronic cGAS-STING activation significantly downregulates TREM2 expression in microglia via pro-inflammatory factors, further exacerbating glial dysfunction [63, 64]. Generally, in the process of NDs, microglial phagocytic activity and pro-inflammatory responses cannot be governed by simple binary roles. Notably, pro-inflammatory and neurotrophic factors can be generated simultaneously. In an in vitro study, researchers found that prolonged activation of microglia produces neurotoxic factors such as ROS and TNF-α, while early activation upregulates brain-derived neurotrophic factor (BDNF) [65].

At the DAM state, microglia clear PPAs via highly active phagocytic mechanism. Single-cell transcriptomics has further revealed stratified gene expression differences between DAMs and homeostatic microglia, with DAMs exhibiting enhanced phagocytic-lysosomal function, oxidative phosphorylation, and antigen-presenting capacity [66, 67]. However, the DAMs simultaneously release proinflammatory cytokines (e.g., interleukin [IL]-1β, TNF-α, and IL-6) and ROS and activate the complement system. It may also propagate protein aggregates via extracellular vesicles, thereby accelerating pathological progression and neurodegeneration [22]. Within neuroinflammatory environments, DAMs can activate the NLRP3 pathway, which is particularly prominent in AD and PD [68, 69]. Additionally, DAM-mediated immune regulation is reflected in its ability to secrete chemokines (e.g., C-C motif chemokine ligand 5 and C-X-C motif chemokine ligand 10) to recruit peripheral immune cells into the CNS. Meanwhile, enhanced antigen presentation by DAMs may serve as a bridge for neuroimmune crosstalk.

Phagocytosis is a multistep process with unclear benefit. Its efficacy depends on the metabolic state and lysosomal function of microglia. Emerging evidence highlights upregulated lysosomal and lipid-handling genes in microglia in NDs. While this adaptation can promote the clearance of pathological proteins, excessive stress may lead to lysosomal dysfunction, lipid accumulation, and secondary release of proinflammatory mediators [70–73]. Enhancing lysosomal function or TREM2 signaling compacts plaques and reduces neuritic dystrophy [74], whereas complement-mediated pruning drives early synaptic loss before obvious neuronal death [75]. This illustrates how subtle shifts in phagocytic targeting alter disease trajectories. When the phagocytosed protein aggregates cannot be cleared, glial cells will rely on exocytosis, exosomes, and TNTs to spread these proteins to other cells. The phagocytosis of α-syn PFFs is TREM2-dependent and aggravates PD pathology. Following phagocytosis, the aggregated α-syn is further cleaved by lysosomal AEP (asparagine endopeptidase) into α-syn 1–103 aggregates, thereby promoting α-syn-related pathology [76].

Given the dual protective and detrimental roles of DAM, studies have proposed precise modulation of microglial phenotypes to promote a transition toward an anti-inflammatory, clearance-oriented protective state. For example, LXRα/β (liver X receptor α/β) agonists or Kv1.3 blockers can promote anti-inflammatory DAM while suppressing proinflammatory DAM, thereby improving the Aβ clearance efficiency. Combinatorial therapies targeting NLRP3 inhibitors also show potential in mitigating DAM-driven inflammation [77, 78]. Analysis of the Human Microglial Atlas (HUmicA) indicates that the DAM subtypes in the human brain are far more complex than those in mouse models. Four distinct DAM or pathological inflammatory microglial subpopulations have been identified, with the GPNMB (Glycoprotein Nonmetastatic Melanoma Protein B)-high subtype significantly expanded in AD and multiple sclerosis, suggesting disease-specific functional adaptations [79].

Immunomodulation by microglia is highly context-dependent. Microglia are sources and recipients of cytokine signals that either resolve damage (e.g., IL-10 and transforming growth factor beta) or amplify injury (e.g., IL-1β and TNF-α). Interestingly, recent interventional studies in rodent models have shown that targeting the immunoregulatory axis has detectable effects. Microglia-directed IL-10 gene delivery reduces neuroinflammation and neuronal loss in a PD model [80], while selective modulation of TREM2 signaling in AD models alters microglial proliferation, plaque architecture and biomarkers of neural injury [71, 72, 81]. Conversely, experimental partial or transient depletion of microglia by CSF1R (colony-stimulating factor 1 receptor) inhibition has produced mixed outcomes across models, ameliorating α-syn spread in some experiments [82, 83] while providing invalid or even worsening outcomes in others [84, 85]. This delineates that the timing, dosing, and disease context are critical for determining whether reducing microglial numbers or altering phenotypes will help or not. Therefore, therapeutic modulation must consider the dual roles of microglia, aiming to restore balanced clearance and immune resolution rather than simply “suppressing” microglial function.

**Fig. 1:**
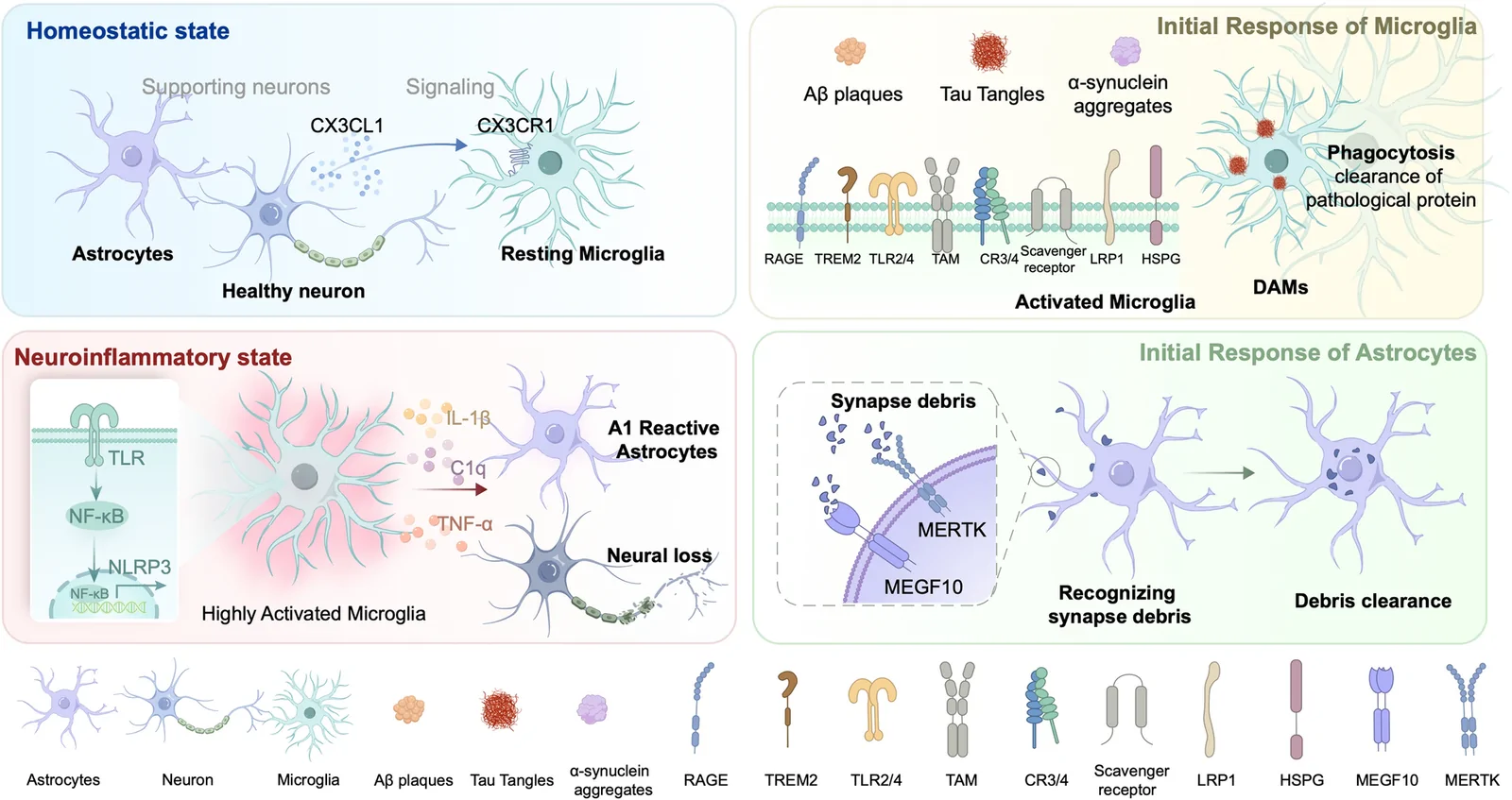
Double-edged roles of glial cells and connections between cells. Microglia serve as both the “guardians” of the central nervous system and, under certain conditions, can transition into "destructors". The overall impact is determined by the dynamic balance between their protective and detrimental functional states. In the early stages of neuropathological insult, resting microglia detect disease-associated molecular patterns, including neuropathological proteins (NAMPs), via receptors such as TREM2. Upon activation, microglia differentiate into disease-associated microglia (DAMs). In this state, they play a neuroprotective role by actively phagocytosing pathological protein aggregates and synaptic debris, thereby contributing to the maintenance of CNS homeostasis. However, under persistent or excessive stimulation, DAMs may undergo a functional shift toward a proinflammatory phenotype. This transition involves the activation of the NLRP3 inflammasome pathway, leading to robust secretion of proinflammatory cytokines. Although this inflammatory response is initially aimed at eliminating pathological threats, its chronic or exaggerated activation results in neuronal toxicity, synaptic dysfunction, and potential exacerbation of pathological protein propagation. Astrocytes can recognize and engulf synaptic fragments through MEGF10 and MERTK, thereby clearing them. Moreover, astrocytes can enhance the clearance by microglia. However, the proinflammatory factors produced by astrocytes under stress conditions can also cause synapse loss

Although disease-specific evidence from recent years has advanced our understanding of how the general mechanisms are manifested in AD, PD, FTLD, and ALS, several questions remain open in the field, such as the precise temporal window for intervention, cell-specific and region-specific delivery in the human brain, and development of fluid and imaging biomarkers that report on microglial functional states rather than simple activation (Fig. 1).

### Astrocytes: maintenance, recognition, phagocytosis, and degradation

Astrocytes have long been regarded as supportive cells in the CNS. However, recent studies have revealed that they play crucial roles in developmental synaptic pruning, damage clearance, and NDs [86–89]. The mechanisms of astrocytic phagocytosis of PPAs are similar to those of microglia. However, the phagocytic capacity of astrocytes is very limited, so they are often regarded to play a supplementary role in phagocytosis while participating more in maintaining homeostasis in the brain. A fundamental research conducted in 2010 provides robust evidence that astrocytes can spontaneously uptake α-syn from neurons, and that astroglial α-syn pathology can originate from neuronal α-syn transfer [90].

Astrocytic recognition of targets relies not only on intrinsic phagocytic receptors but also on humoral components and signals derived from other glial cells. Among these, multiple epidermal growth factor-like domains protein 10 (MEGF10) and MERTK have been identified as key receptors that enable astrocytes to recognize and phagocytose synaptic debris and cellular remnants [91]. Intriguingly, MEGF10 is a transmembrane receptor that is mainly expressed in astrocytes. It can bind to “eat-me” signals such as C1q and drive the clearance of apoptotic neurons in astrocytes [92]. It can also directly promote the uptake of Aβ [93]. Upon recognition of apoptotic cells or other engulfment targets, MEGF10 signals through its intracellular NPxY motif to recruit the adaptor protein engulfment adaptor PTB domain-containing protein 1 (GULP1), thereby initiating a conserved engulfment cascade. GULP1 subsequently promotes the Rac1-dependent actin cytoskeletal remodeling, leading to phagocytic cup formation and target internalization. Functionally, this pathway coordinates with ATP Binding Cassette Subfamily A Member 1 (ABCA1), which facilitates efficient engulfment, although the precise molecular interaction between ABCA1 and MEGF10 remains incompletely understood [94, 95]. Studies have also shown that, in collaboration with MERTK, MEGF10 participates in astrocytic clearance of synapses in the developing and adult brains [91]. Studies using genetic knockout models and in vitro experiments have demonstrated that the loss of these receptors leads to synaptic accumulation and plasticity deficits, confirming the direct role of astrocytes in synaptic elimination [96].

Astrocytes can also influence the microglial phagocytic activity by secreting or modulating complement components, thereby establishing complementary or compensatory clearance mechanisms within the local microenvironment [97]. Under certain neuropathological conditions, such as AD pathology or impaired microglial function, the phagocytic activity of astrocytes is increased and contributes to pathological synapse loss. These findings suggest that while astrocytes help maintain homeostasis, they may also act as drivers of damage in disease contexts [98–101].

Mechanistically, astrocytic phagocytosis employs diverse and context-dependent cellular pathways. Astrocytes engulf larger debris through classical phagocytosis and utilize non-canonical autophagy-related pathways such as LC3-associated phagocytosis (LAP) to process engulfed materials [102, 103]. Recent studies have shown that astrocytes can recognize and phagocytose microglial debris in vivo, a process facilitated by C4b, followed by degradation via a RUBICON-dependent LAP pathway [103]. After cells recognize and phagocytose targets via surface receptors, RUBICON is recruited to the phagosomal membrane, where it assembles the LAP-specific PI3KC3 (class III phosphatidylinositol 3-kinase) complex, which catalyzes the production of phosphatidylinositol-3-phosphate (PI3P). Subsequently, RUBICON stabilizes the NADPH oxidase 2 complex on the phagosomal membrane through direct interaction, promoting its sustained production of ROS. ROS and PI3P together recruit the LC3 lipidation system, catalyzing the lipidation of LC3-I to LC3-II and covalently conjugating it to the phagosomal membrane to form LAPosomes. Finally, the presence of LC3-II significantly accelerates the fusion of LAPosomes with lysosomes, enabling efficient degradation of phagocytosed contents. These findings highlight the role of astrocytes in maintaining population homeostasis and limiting the spread of inflammation. Moreover, the lysosomal function is critical for the degradative capacity of astrocytes. Impaired lysosomal acidification leads to the intracellular accumulation of phagocytosed material and triggers neuroinflammation, providing a rationale for therapeutic strategies targeting lysosomal or autophagy regulation.

Under pathological conditions, astrocytic functions are modulated by inflammatory cytokines, hormones, and the local complement system. Inflammatory cytokines such as TNF-α, IL-1, and IL-6, serve as central regulators of immune responses in the CNS. In NDs, they activate astrocytes, inducing a proinflammatory phenotype characterized by increased secretion of proinflammatory cytokines and chemokines. This activation not only exacerbates local inflammatory responses, but may also contribute to neuronal injury and dysfunction [104]. Hormones also modulate astrocytic function. Astrocytes express multiple subtypes of estrogen receptor. Estrogen regulates gene expression in astrocytes by activating its receptors on astrocytes, facilitating the expression of various growth factors, which manifest a neural supportive phenotype [105, 106]. In NDs, alterations in hormone levels may modify astrocyte reactivity, thereby potentially affecting disease progression [104, 107]. Local complement system activation represents another crucial regulatory mechanism. C3 and C4 have been implicated in astrocyte activation in the neurodegenerative context [108]. For example, the complement C4 amplifies inflammatory responses in astrocytes, leading to increased neuronal apoptosis and synaptic damage in α-syn PFF-injected mice. C4 also facilitates the propagation of α-syn pathology and synaptic loss in cultured neurons [109]. Conversely, enhancing lysosomal acidification or correcting the LAP function in astrocytes may represent a promising neuroprotective strategy [110].

Increasing evidence indicates that astrocytes exhibit remarkable heterogeneity at morphological, molecular, and functional levels, challenging the traditional view that they represent a homogeneous glial population. Single-cell and spatial transcriptomic analyses have demonstrated that astrocytes display pronounced regional specialization across the CNS, with distinct gene expression programs in the cortex, hippocampus, striatum, cerebellum, and white matter. Even within the same anatomical region, multiple astrocyte subpopulations coexist and differ in the expression of genes involved in neurotransmitter recycling, lipid metabolism, synaptic regulation, phagocytosis, immune signaling, and extracellular matrix remodeling [111, 112]. Importantly, accumulating evidence suggests that astrocytes adopt disease-specific reactive states rather than a single universal activation phenotype. Some subsets are enriched for inflammatory mediators and complement components, including C3, and cytokine-responsive genes, whereas others preferentially express lysosomal, lipid metabolic, stress-response, or phagocytosis-related programs involving genes such as *GPNMB*, *CTSB*, *LAMP1*, and *APOE*. Furthermore, these transcriptional and functional states are highly dynamic and evolve throughout disease progression in response to local microenvironment, necessiting the consideration of astrocyte heterogeneity when investigating glial-mediated protein clearance and designing therapeutic strategies [113]. Also, some pathways involved in protein clearance and neuroinflammation may not be conserved across species. A study found extensive conservation in the overall transcriptome between human and mouse astrocytes, but there are also species-specific differences in genes involved in defense response and metabolism. In the resting state, mouse astrocytes show high enrichment of mitochondrial and metabolism-related genes, while human astrocytes express more genes related to defense and immunity. When exposed to oxidative stress, mouse astrocytes show high expression of antioxidant detoxification genes, resulting in more stable mitochondrial function and stronger tolerance, while human cells are more prone to damage. In hypoxic environments, mouse astrocytes will initiate transcriptional programs related to neural repair, while human astrocytes do not respond. Under inflammatory or viral stimulation, human astrocytes will significantly activate MHC I (major histocompatibility complex class I) antigen presentation and inflammatory pathways, while mouse astrocytes show a weak response. In addition, the transcriptional characteristics induced by inflammation in human astrocytes are highly consistent with the disease‑associated astrocyte states in human pathology, while there is no obvious association in mice. This also explains the poor translational effect of mouse neural disease models in clinical applications [114].

Thus, astrocytes exhibit complex and highly context-dependent biological behaviors. Future research is needed to elucidate the different functions of astrocyte subtypes, clarify how intercellular signals are spatiotemporally coordinated, and selectively modulate astrocytic clearance and repair functions without disrupting normal synaptic plasticity. Such insights may provide new targets and strategies for the diagnosis and treatment of NDs.

### The UPS and autophagy-lysosome pathway

**Fig. 2:**
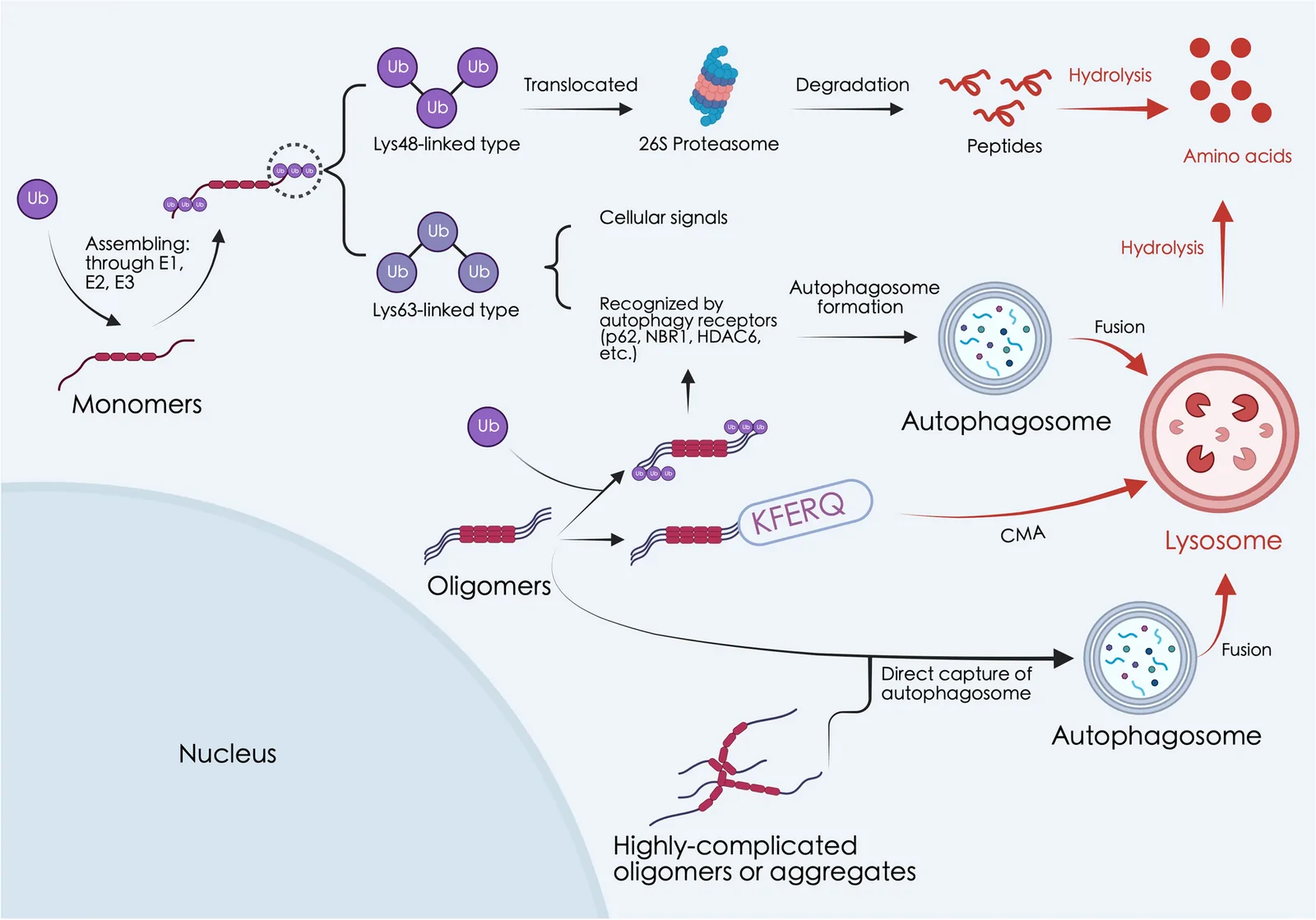
Clearance pathways of pathological proteins. In neurons and other cell types, the ubiquitin-mediated proteasomal system (UPS) and chaperone-mediated autophagy (CMA) can remove monomers and partial oligomers, while highly misfolded proteins are removed through direct capture by the autophagosome or lysosome. Proteins destined for the proteasome are polyubiquitinated and guided to the proteasome to be degraded. CMA acts on proteins with KFERQ-like sequences. Heat shock protein 70 (HSC70) recognizes the KFERQ sequence and transports the protein together with chaperones to the LAMP2A receptor, which is located in lysosomes. LAMP2A then coordinates with protein transport into the lumen. Autophagosomes capture highly misfolded, high-molecular-weight, aggregated proteins and damaged organelles and carry cargo to fuse with acidic lysosomes, resulting in degradation of their contents. Moreover, different ubiquitination linkage patterns lead to different outcomes. Proteins with Lys48 linkages are mainly degraded by proteases, while proteins with Lys63 linkages are mainly recognized and engulfed by autophagosomes. Figure created with Biorender.com

The UPS serves as a highly specific, energy-dependent mechanism to degrade short-lived, misfolded, or damaged proteins. The substrates are tagged by ubiquitin through a cascade of E1, E2, and E3 enzymes. During protein ubiquitination, differences in the chain linkage patterns of multiple ubiquitin molecules lead to distinct inter-ubiquitin linkage sites, thereby tagging ubiquitinated proteins with unique features. These features ultimately determine that ubiquitinated proteins are degraded through two distinct pathways: proteasomal hydrolysis and autophagy. Ubiquitin molecules contain seven lysine (Lys) residues and one N-terminal methionine (Met), all of which can serve as sites for polyubiquitin chain formation. Common linkage modes include Lys48-linked polyubiquitin chains, which is the most classical and well-studied mode and typically serves as a “death signal” marking substrate proteins for degradation by the 26S proteasome; and Lys63-linked polyubiquitin chains, which are not involved in degradation but participate in cellular signaling processes such as the DNA damage response, multivesicular body sorting, NF-κB signaling, and oxidative stress responses. Notably, the Lys63-linked chain spontaneously acts as an autophagy recognition signal, recognizing autophagy receptors, including p62/SQSTM1, NBR1, and HDAC6. Other atypical linkage modes, including Lys6, Lys11, Lys27, Lys29, Lys33, and Met1 chains, often perform non-degradative functions, such as regulating cell cycle progression, protein complex conformation, or signaling pathways [115–119]. There are also mixed or branched linkage chains, such as Lys29/Lys48 or Lys11/Lys63 mixed chains, which play critical roles in modulating diverse cellular processes, including protein degradation and signal activation [117] (Fig. 2).

Based on the distinct mechanisms of cargo delivery and membrane dynamics, autophagy is systematically categorized into three primary pathways: macroautophagy, microautophagy, and CMA [120–122]. Macroautophagy involves the elongation of isolation membranes to form double-membraned autophagosomes that sequester cytoplasmic components, which range from bulk cellular contents to specific substrates, before fusing with lysosomes for degradation. In contrast, microautophagy involves the direct invagination of lysosomal or endosomal membranes to generate intraluminal vesicles that encapsulate cytosolic material for lysosomal breakdown. Additionally, endosomal microautophagy (eMI) facilitates the extracellular release of a subset of these vesicles as exosomes. CMA involves the recognition of the KFERQ motif by the chaperone HSC70 (heat shock cognate 70 kDa protein), which forms complexes that dock to the lysosomal receptor lysosome-associated membrane glycoprotein 2 (LAMP2A) for direct translocation and degradation within lysosomes, which is highly specific.

Despite conventional views that the UPS and autophagy are independent systems, increasing evidence supports their integration into a network. Typically, autophagy receptors such as p62/SQSTM1 bind polyubiquitinated substrates and deliver them to proteasomes or to autophagosomes via interactions with LC3. Intriguingly, inhibition of proteasomes leads to compensatory activation of autophagy. Proteasomal stress and the subsequent accumulation of misfolded proteins trigger signaling pathways involving PERK-eIF2α-ATF4-CHOP and IRE1-TRAF2-JNK, increasing the expression of autophagy genes and initiating autophagosome biogenesis [123–125]. Conversely, excessive autophagy can inhibit the UPS function. Typically, soluble monomers are substrates of proteases, whereas insoluble PPAs are primarily degraded through autophagy [126]. Thus, impaired autophagy leads to increased PPAs [125, 127, 128]. Notably, during mitophagy, novel protein complexes containing autophagy protein ATG5 and UPS components PSMA7 and PSMB5 regulate Parkin (PARK2) recruitment to mitochondria, indicating direct collaboration between these two systems [129].

In patients with NDs, early-stage soluble proteins or peptides initially bind to E3 ubiquitin ligases (e.g., CHIP, TRAF6, axotrophin/MARCH7, HRD1, and UL5), leading to their ubiquitination and subsequent targeting for degradation via the 26S proteasome. When the UPS is impaired or overwhelmed beyond its clearance capacity, pathological proteins tend to aggregate. Low-aggregation pathological proteins can be cleared through microautophagy or CMA. In contrast, highly aggregated, insoluble PPAs are degraded primarily via macroautophagy. Enhancing the UPS and autophagy pathways can effectively promote the clearance of pathological proteins.

### Cell–cell interactions between neurons and glial cells

The nervous system is a highly interconnected cellular network. In addition to synaptic connections and long-range axonal projections between neurons, various glial cells engage in continuous bidirectional communication with neurons through direct contact, chemical signaling, extracellular vesicles, membrane bridges, and other pathways. These cellular interactions not only maintain normal developmental and functional homeostasis, but also contribute to pathological propagation or lesion clearance under pathological conditions.

Synaptic pruning during development is a classic example of neuron-glial interaction. Under normal conditions, microglia and astrocytes identify and eliminate redundant or weakened synapses, thereby refining neural circuits. This process relies both on complement-mediated tagging of synapses destined for elimination (e.g., via the C1q/C3 pathway) and on direct phagocytic receptors such as MEGF10 and MERTK expressed on astrocytes, which mediate synaptic engulfment [91, 100]. These mechanisms have been functionally validated in animal models, demonstrating that glial cells achieve selective synaptic removal through coordinated receptor‒ligand interactions and immune-related tagging.

In the adult brain, communications between neurons and glial cells become more diverse and dynamic. Astrocytes sense local neural activity through their perisynaptic processes and regulate neurotransmitter clearance, energy metabolism, and ion homeostasis, thereby influencing synaptic plasticity and excitability balance [130, 131]. Moreover, microglia continuously monitor the microenvironment, rapidly extending their processes to “sniff” synaptic surfaces [22]. Upon detection of damage or pathological signals, they may perform phagocytosis or release inflammatory mediators, ultimately affecting neuronal survival and function in complex and contradictory ways.

Significant crosstalk also occurs among glial cells. Cytokines and complement components produced by microglia can induce phenotypic shifts in astrocytes, increasing their phagocytic activity or stimulating the secretion of specific exosomes under certain pathological conditions. Conversely, metabolic products and regulatory secretions from astrocytes can shape the developmental states and response profiles of microglia. Such interactions are particularly critical in brain injury, chronic inflammation, and aging. When one type of glial cell is impaired, another may partially compensate. This effect might be supportive. Nonetheless, it can also lead to aberrant amplification of inflammatory or phagocytic responses, ultimately impairing the neural circuit function [132].

In NDs, dysfunction at any node of the cell-to-cell system, whether complement-mediated excessive synaptic loss, impaired lysosomal clearance leading to accumulation of pathological proteins, or toxic intercellular spread via EVs, can drive disease onset or progression. Therefore, future therapeutic strategies should not only target protein folding and metabolic abnormalities within neurons, but also restore or modulate glial phagocytosis and degradation capacity, regulate EV biogenesis and uptake, and rebalance the glia-glia and glia-neuron interactions, in order to simultaneously inhibit pathological spread of PPAs and preserve synaptic plasticity.

### Trafficking of pathological proteins in glial clearance: roles of EVs and TNTs

EVs serve as an important route for material and signal transfer between neurons and glial cells. Neurons, astrocytes, and microglia all secrete EVs containing proteins, nucleic acids, and lipids. These vesicles can be taken up by neighboring or distant cells, transmitting metabolic signals, stress messages, or pathological proteins. In NDs, EVs act as a double-edged sword. On the one hand, they may facilitate waste clearance or serve as warning signals, helping surrounding cells clear toxic substances and activating repair pathways. On the other hand, EVs carrying pathologically misfolded proteins can promote intercellular spread of pathology. Studies of both human brain-derived EVs and animal models indicate that EV compositions are altered under disease conditions and that some glia-derived EVs are more readily taken up by neurons, potentially serving as vehicles for protein “propagation” and neurotoxicity [133, 134].

Newly discovered TNTs also contribute to intercellular transport and clearance of PPAs. Astrocytes can receive and degrade autophagosomes or protein aggregate-containing vesicles derived from neurons through TNTs. Conversely, under cellular stress, astrocytes may transfer mitochondria or other organelles to neighboring cells to support their metabolism [135–137]. Lysosomal function, autophagy pathways, and molecules such as RUBICON play central roles in regulating these processes. Dysfunction of these regulators leads to the accumulation of phagocytosed material, triggering inflammatory responses or cellular failure, thereby accelerating neurodegeneration.

### Lymphatic clearance of PPAs

**Fig. 3:**
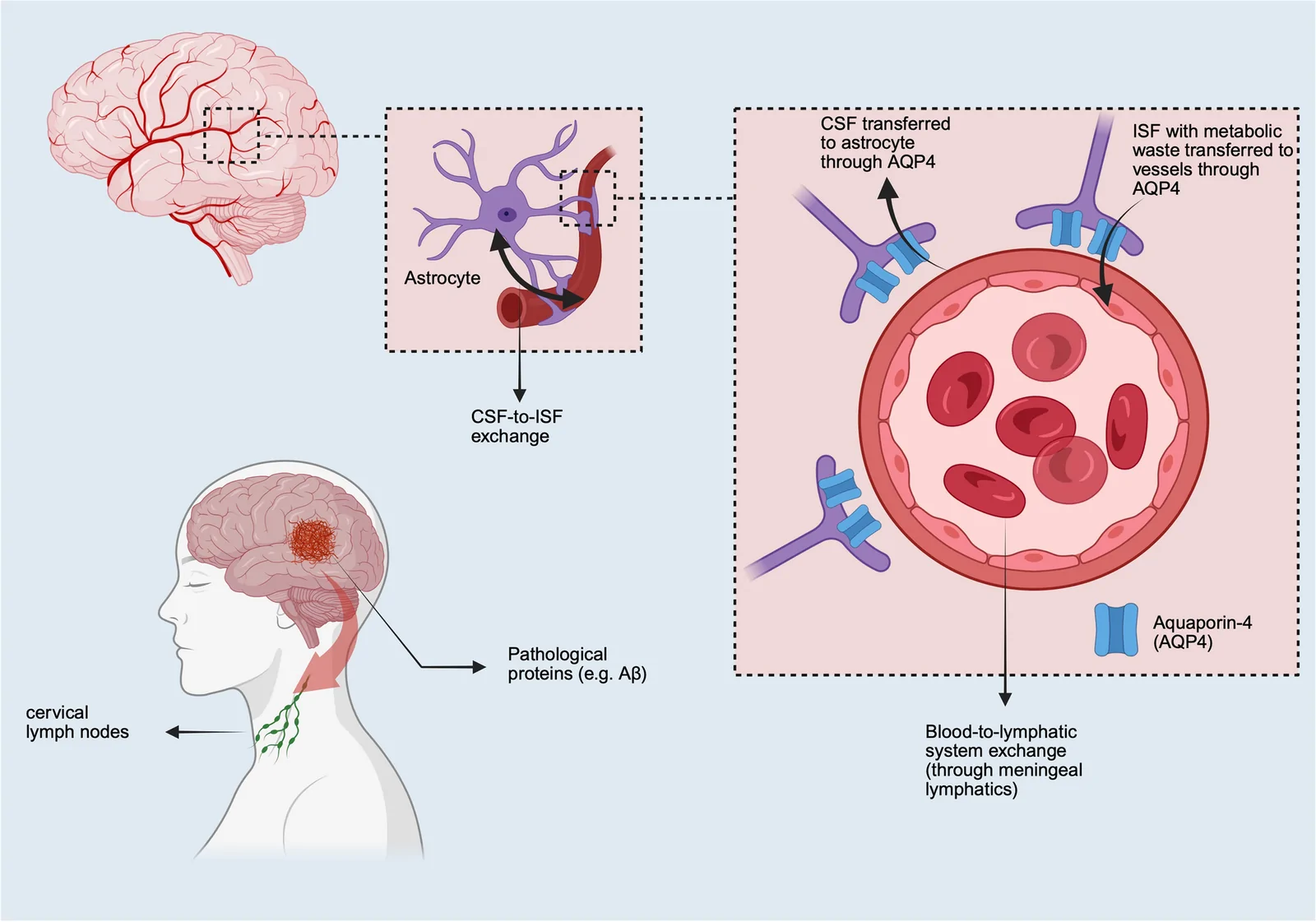
Roles of the lymphatic system in protein clearance. The brain clears metabolic waste through a complex set of lymph-related pathways. The core of this process is the collaboration between the glymphatic system and the meningeal lymphatic vessels. The glymphatic system drives cerebrospinal fluid (CSF) to flow into the brain parenchyma along the perivascular spaces of the arteries, where it exchanges waste with the interstitial fluid (ISF), and the mixed fluid carrying solutes (such as Aβ and tau proteins) flows out along the perivascular spaces of the veins. The flowing-out fluid is then captured by the meningeal lymphatic vessels and eventually drained to the cervical lymph nodes, thereby discharging the waste of the central nervous system into the peripheral processing system. During this process, aquaporin 4 (AQP4) is expressed on the footplates of astrocytes, serving as a key “valve” for driving efficient fluid exchange and waste clearance between CSF and ISF. Figure created with Biorender.com

Macroscopically, the clearance of waste and abnormal proteins in the brain relies on an integrated fluid‒lymphatic coupled system. The glymphatic pathway facilitates the exchange between cerebrospinal fluid (CSF) and interstitial fluid (ISF), transporting soluble metabolites and pathological proteins from the brain parenchyma toward the brain surface [138–141]. Moreover, recently discovered meningeal lymphatic vessels capture this efflux fluid and ultimately drain it into the cervical lymph nodes, thereby connecting to peripheral processing networks. Aquaporin-4 (AQP4), a water channel protein localized at the endfeet of astrocytes, plays a central role in maintaining influx capacity at the CSF-to-ISF interface and driving fluid exchange [142, 143]. AQP4 is highly polarized to the perivascular endfeet of astrocytes surrounding arteries, enabling CSF to enter the parenchyma along periarterial spaces and infiltrate the interstitial compartment through this polarized astrocytic network [144]. Simultaneously, AQP4 allows metabolic waste and soluble pathological proteins within the ISF to efflux via analogous channels toward the brain surface or perivenous spaces, where they are captured by meningeal lymphatics and cleared through cervical lymph nodes (Fig. 3).

Under physiological conditions, this coupled system operates efficiently. Arterial pulsation, sleep, vascular compliance, and structural integrity of astrocytic endfeet and the vascular basement membrane collectively ensure the polarized localization of AQP4 and unimpeded fluid flow. The rate of CSF-to-ISF exchange fluctuates significantly with sleep–wake cycles. The clearance efficiency is enhanced during sleep and diminished during wakefulness or sleep deprivation [145, 146]. The localization and the expression of AQP4 are tightly regulated throughout this process to optimize efficiency during both CSF influx and waste efflux. Meningeal lymphatic vessels drain fluid into cervical lymph nodes for degradation by the peripheral immune system and other degradation mechanisms. The drainage can be modulated by intracranial pressure fluctuations and cardiorespiratory activity.

However, conditions such as aging, presence of genetic risks such as *APOE4*, sleep disturbances, decreased cerebrovascular function, structural impairment of astrocytes or vascular support cells, and inflammatory responses, can disrupt the polarized state of AQP4. Loss of polarization typically manifests as a redistribution of AQP4 away from perivascular endfeet to other fine processes or nonendfoot regions of astrocytes. This mislocalization impedes the rapid influx of CSF along periarterial spaces into the parenchyma and slows the efflux of pathological proteins from the ISF. Pathological proteins such as Aβ, α-syn, and tau, which are efficiently cleared in wild-type models, accumulate more rapidly in AQP4-depleted or deficient models. Furthermore, meningeal lymphatic function may decline due to the lymph vessel density. When the meningeal lymphatic density decreases, vessel elasticity and integrity are compromised, or the fluid supply at the influx sites is insufficient. Therefore, the drainage capacity to cervical lymph nodes is impaired, even if glymphatic inflow remains intact. This dual mechanism of reduced efficiency of AQP4-driven influx and decreased meningeal lymphatic outflow collectively lead to impairment of pathological protein clearance. Concurrently, the load captured by meningeal lymphatics decreases, leading to disruption or obstruction of the entire drainage pathway to the cervical lymph nodes [143, 147].

In summary, the integrated glymphatic–meningeal lymphatic–cervical lymphatic drainage pathway, coupled with the polarized localization of AQP4, constitutes a complete fluid system. Dysregulation at any node can compromise the overall clearance efficiency of the pathway, leading to accumulation of pathological proteins in the brain. This provides insights into potential interventional strategies, such as protecting or restoring AQP4 polarization, improving cerebrovascular and lymphatic structure, and optimizing sleep and vascular dynamics to enhance the overall clearance function of the system.

### Clearance of pathological proteins in NDs

During the development of AD, the phenotypes and receptor expression of microglia and astrocytes undergo dynamic changes, which are closely linked to Aβ and tau pathologies and affect the progression of the disease. As Aβ plaques and tau neurofibrillary tangles accumulate, microglia shift from a stable state to an activated phenotype, exhibiting abnormal expression or functions of multiple receptor pathways. For instance, TREM2 is typically upregulated in AD pathological regions, but its activation declines with the progression of the disease, which is related to the weakened phagocytic clearance ability of microglia toward Aβ [23]. The ITAM signaling pathway formed by TREM2 and its adapter protein DAP12 responds to the accumulation of pathological proteins. Functional defects of this pathway will aggravate the disease. The TREM2 risk variant (such as R47H) leads to impaired ITAM signal transduction, thereby affecting microglial response to Aβ [148]. In addition, the inhibitory receptor CD33 is significantly overexpressed in AD microglia, and its ITIM signal can negatively regulate the phagocytic function of microglia and inhibit the downstream clearance pathway. In the APP/PS1 model, CD33 deficiency improves Aβ pathology, and this effect depends on the presence of TREM2, indicating the interactions of the two pathways [149]. In the APP/PS1 model, microglial Axl and MERTK (two TAM receptors) are strongly upregulated around Aβ plaques. Deletion of Axl and MERTK dramatically impairs microglial plaque recognition and engulfment, suggesting that TAM receptors are essential mediators of microglial phagocytosis of amyloid aggregates in vivo [46].

PRRs (such as TLR2/4) and the NLRP3 inflammasome are generally overexpressed in AD. After Aβ binds to these receptors, microglia and astrocytes activate NF-κB and NLRP3 inflammation cascades, releasing pro-inflammatory factors such as IL-1β and TNF-α, which promote chronic inflammation and exacerbate neuronal damage. TLRs also cross-activate the complement system. Aβ can simultaneously trigger TLR4 and the complement cascade, promoting microglial phagocytosis but also leading to excessive synaptic clearance and persistent inflammation [150, 151]. CRs (such as CR4) are upregulated in AD, where they can recognize tau and Aβ fibers and promote their clearance [60, 61]. However, excessive complement activation is also associated with non-specific synaptic pruning and inflammatory damage, acting as a potential pathological amplifier [101].

The expression of LRP1 in astrocytes is reduced in AD, decreasing the ability to clear Aβ. The damaged astrocytic LRP1 may also weaken blood–brain barrier (BBB) homeostasis [152, 153]. HSPGs can mediate the binding and endocytosis of Aβ or tau, but their specific expression and glycosylation status vary across different brain regions and disease stages, which may affect the cellular uptake and diffusion of pathological proteins [154–156]. In addition, scavenger receptors (such as CD36) are involved in Aβ endocytosis under AD pathology, which exhibits TREM2-dependency [157], but they can simultaneously promote pro-inflammatory signals with TLR4/TLR6 [158].

The core pathology of PD is the formation and spread of α-syn aggregates. Glial cells undergo functional alterations, characterized by impaired phagocytosis and clearance of pathological proteins, alongside elevated inflammatory activity and cellular cytotoxicity. α-Syn fibrils and oligomers bind to TLR2/TLR4 on microglia, activating the NF-κB pathway and the NLRP3 inflammasome, leading to the release of pro-inflammatory factors such as IL-1β/IL-18. This results in a chronic inflammatory state and promotes the degeneration of dopaminergic neurons [159–161]. In PD, microglial activation is often accompanied by a DAM-like phenotype, similar to AD, but the specific receptor expression pattern shows α-syn dominance, such as activation of TLR2, leading to continuous activation of NF-κB and MAPK [161–163].

TREM2 exhibits a complex dynamic pattern in PD. The expression of TREM2 is decreased in PD patients and models [164, 165]. Absence of TREM2 is associated with enhanced inflammatory responses in microglia (increased levels of NLRP3, IL-1β, and IL-18) and more severe loss of dopaminergic neurons [166, 167]. Although CD33 has been less studied in PD, its intracellular ITIM domain mediates inhibitory signaling upon ligand ligation, suppressing microglial phagocytic activity, which may contribute to pathological α-Syn accumulation. Similar to the negative regulation of phagocytosis described in AD, this CD33-ITIM inhibitory axis warrants further investigation in PD. Additionally, MERTK and its ligands (GAS6/PROS1) are important for microglial processing of α‑syn pathology, linking TAM receptors to both synucleinopathies and amyloidopathies [48].

In PD pathology, the complement system and FcγRs are also involved in the recognition of pathological proteins and the amplification of inflammation. α-Syn aggregation can induce complement activation and be recognized by microglia CRs for clearance. However, excessive activation of the complement cascade may promote an imbalance in inflammation and clearance. Among FcγRs, the α-syn-antibody complex can activate microglia through FcγRs, enhancing the inflammatory response and possibly damaging adjacent neurons [109]. LRP1 is involved in the internalization and spread of α-syn in PD. The specific expression and function of LRP1 vary depending on the disease course. Generally, the LRP1-mediated endocytic pathways are activated in glia in the presence of α-syn aggregates, but this may be accompanied by high load-induced cellular stress [168–170].

In glial cells in PD, PRR signaling examplified by TLR and NLRP3 pathways serves as a key pathological driver. Activation of TLR/NLRP3 signaling in microglia triggers crosstalk with astrocytes, amplifying local inflammatory responses. Together with synaptic dysfunction and mitochondrial dyshomeostasis, this process drives neurodegenerative alterations [161]. HSPG may mediate the endocytosis and propagation of α-syn, but the exact role still requires further research [171]. Notably, different mutations in glia exhibit different responses and phagocytic functions toward PPAs. Results from transmission electron microscopy and proteomics analysis revealed that astrocytes harboring the leucine-rich repeat kinase 2 (*LRRK2*) G2019S mutation exhibit significantly reduced efficiency in endosomal-lysosomal uptake and degradation of α-syn aggregates compared to wild-type astrocytes. This impairment is attributed to the loss of Annexin A2 (AnxA2) function. This further suggests that LRRK2 in astrocytes participates in the clearance of extracellular α-syn aggregates through an AnxA2-dependent mechanism [172].

## Therapeutic strategies targeting the clearance of PPAs

Current therapeutic strategies for major NDs uniformly focus on restoring neuronal protein homeostasis via targeted clearance of disease-specific misfolded and aggregated pathological proteins. The core pharmacological approaches broadly encompass immunotherapies that mediate microglial phagocytosis and peripheral/central elimination of toxic protein aggregates, protein-targeted interventions and small molecules that inhibit the synthesis and maturation of aberrant protein species, molecular chaperones that correct abnormal protein folding to block aggregate formation, and pharmacological activators of the autophagy-lysosome pathway to potentiate intracellular degradation and clearance of pathological protein inclusions. All these therapeutics aim to mitigate neurotoxicity, arrest progressive neuronal damage, and achieve fundamental disease modification in neurodegenerative conditions.

### Aβ and tau in AD

A most recent review summarized that 138 drugs for AD treatment are currently undergoing 182 clinical trials. These candidate drugs span multiple therapeutic areas, including Aβ, tau, neuroinflammation, metabolic function, and synaptic function [173]. Among them, Aβ-targeting drugs account for approximately 18% of AD drugs currently in development. Primary strategies include reducing Aβ production (such as the earlier development of β-secretase inhibitors), preventing Aβ aggregation, and promoting the clearance of existing amyloid plaques. Current research efforts have shifted toward the latter two approaches, such as passive immunization with monoclonal antibodies and active immunotherapy vaccines. Lecanemab is a monoclonal antibody that specifically targets Aβ fibrils and oligomers and has been approved by the FDA for the treatment of patients with early-stage AD [174, 175]. Donanemab is also a monoclonal antibody that targets N3pG-modified Aβ, a more toxic form of Aβ in pathological states, and has been approved by the FDA for marketing [176, 177]. Aducanumab is the first Aβ-targeting drug to receive accelerated FDA approval. However, its efficacy remains highly controversial in phase III clinical trials [176, 178], leading to the termination of the project. Lanabecestat is an inhibitor for human β-site amyloid precursor protein cleaving enzyme 1 (BACE1/β-secretase), capable of crossing the BBB. The primary mechanism of lanabecestat involves reducing Aβ production to slow the progression of AD [179]. These strategies primarily aim to reduce Aβ production or deposition. Despite the recent approval of Lecanemab and Donanemab antibodies, the history of Aβ-targeting therapies reveals significant clinical failure rates. For instance, BACE1 inhibitors such as Lanabecestat failed in Phase III trials not due to off-target toxicity, but because chronic inhibition of Aβ production led to compensatory upregulation of non-amyloid pathways or exacerbated synaptic dysfunction. Moreover, many Aβ-targeting antibodies that effectively reduced plaque burden in transgenic mice showed no cognitive benefit in humans, which may be related to the following factors. First, murine models overexpress mutated human amyloid precursor protein under artificial promoters, leading to supraphysiological plaque deposition that does not recapitulate the slow, decades-long human pathology. Second, human cerebrovascular structure and perivascular clearance (including the glymphatic system) differ substantially from mice, contributing to amyloid-related imaging abnormalities as seen with Aducanumab, limiting the dose of use. Third, by the time patients become symptomatic, irreversible neuronal loss has already occurred, whereas antibodies are most effective at removing monomers and oligomers but cannot restore dead neurons.

By early 2025, approximately 11% of AD drugs in development target tau-related pathways. While breakthroughs have been made in the Aβ-targeting field, no successful tau-targeting drugs have yet reached the market. However, given the strong correlation of tau pathology with cognitive impairment, tau-targeting therapies are considered to hold significant potential for achieving important clinical outcomes. Posdinemab is a humanized IgG1 monoclonal antibody that binds to residues 204–225 of tau. This antibody exhibits high affinity for tau pT217. Phase I studies demonstrated that posdinemab reduces CSF levels of total p-tau and p-tau217 in a dose-dependent manner [180]. Currently, posdinemab is in phase II clinical trials (NCT04619420) for early-stage AD patients [181]. Semorinemab is a tau-targeting monoclonal antibody whose core mechanism involves targeting the extracellular tau protein to prevent its pathological propagation between neurons. The drug is currently undergoing phase II clinical trials [182, 183]. AADvac1 is an active vaccine that elicits an immune response to produce antibodies targeting pathological tau proteins. It is currently undergoing a phase II clinical trial [184]. LMTX is an inhibitor for abnormal tau protein aggregation, thereby preventing the formation of neurofibrillary tangles. The drug has advanced to phase III clinical trials and is currently undergoing data analysis [185]. The latest tau-targeting antibodies, such as E2814 and bepranemab, target the microtubule-binding region or its neighboring areas. Some of these antibodies can identify specific p-tau217 pathological forms. The results of the study show that E2814 significantly reduces the CSF level of p-tau217 [186]. Bepranemab improved both tau PET burden and cognitive function for the first time in phase II trials. The effect was more significant in patients with lower baseline tau levels or APOE4 negativity [187]. The failure of tau-targeting therapies to date could be attributed to several translational barriers. First, tau pathology in humans propagates over decades, whereas animal models (e.g., P301S mice) show rapid, supraphysiological spread that does not mirror the seeding process of tau conformers in human brains. Second, most monoclonal antibodies, including Semorinemab, are designed against extracellular tau. However, the majority of pathological tau is intracellular, limiting antibody access unless coupled with Fc-receptor-mediated uptake or neuronal death. Third, active vaccines such as AADvac1 induce variable antibody titers in elderly patients due to the aging of the immune system, and the induced antibodies may recognize truncated or post-translationally modified tau species that are not the dominant pathogenic forms in humans. A notable example is LMTX, a tau aggregation inhibitor that reached Phase III but failed to show benefit over placebo, partly because of off-target binding and inadequate brain penetration in aged humans compared to transgenic mice. Therefore, future tau-directed strategies must validate target engagement in CSF or using PET biomarkers rather than relying solely on mouse behavioral readouts. Future studies of new targets also require attention. Research has found that the presence of pathological tau aggregates activates the TRIM21 ubiquitin ligase function. By fusing the RING domain of the TRIM21 E3 ubiquitin ligase with a specific nanobody to selectively ubiquitinate and degrade aggregated tau through the proteasome, researchers have demonstrated a new paradigm for selective removal of aggregates [188]. While TRIM21-based nanobody degradation and cGAS inhibition show promise in rodent models, their translational potential is limited by the species differences in the UPS and type I interferon (IFN-I) signaling kinetics. Human microglia exhibit lower basal cGAS-STING activity and different interferon-stimulated gene profiles. Thus, these strategies may require validation in human iPSC-derived microglial models before clinical advancement. Research has shown that in the context of Aβ pathology, *APOE3* variants can enhance the reactivity of microglia to clear plaques, including phagocytosis and lysosomal function. This inhibits Aβ-induced tau seeding, suggesting that regulating the state of glial cells is a potential therapeutic strategy [189]. Studies have found that pathological tau protein activates cGAS and IFN-I responses in microglia. The cGAS activation reduces the neuronal transcriptional network of myocyte enhancer factor 2c (MEF2C) through the IFN-I signaling pathway, thereby exacerbating cognitive impairment. Knockout of the cGAS gene in mice with tauopathy reduces the IFN-I response in microglia, maintaining synaptic integrity and plasticity and thus protecting against cognitive impairment. Furthermore, pharmacological inhibition of cGAS enhances the neuronal MEF2C transcriptional network and improves synaptic integrity, representing a new therapeutic target [190]. Tau-targeting therapies aim to reduce the accumulation and propagation of toxic tau. Monoclonal antibodies reduce pathological spread by binding to extracellular tau or engulfing tau seeds. Small molecules maintain normal tau conformation by interfering with posttranslational modifications or directly blocking aggregation. Vaccines induce sustained immune surveillance targeting tau in vivo. Given the complexity of tau-related pathways, single interventions may require extended time to show clinical efficacy. Current tau-targeting therapies remain exploratory. In the future, they can potentially be combined with other therapies to comprehensively address AD pathology.

In addition to the above targets, microglial receptors and their downstream signaling pathways have emerged as important modulators of AD progression. Notably, TREM2 agonists have been shown to engage the SYK/AKT/ERK/mTOR signaling cascade, enhancing microglial phagocytic clearance of Aβ and attenuating excessive phosphorylation of endogenous tau. By simultaneously promoting pathological protein removal and mitigating tau hyperphosphorylation, TREM2 activation may therefore slow the progression of AD [191, 192]. Recent evidence indicates that activation of innate immune sensors such as the cGAS-STING and the NLRs pathways contributes to microglial inflammation in AD. Pharmacological inhibition of STING signaling has been shown to attenuate microglial dysfunction, reduce Aβ deposition and tau phosphorylation, and ameliorate cognitive decline in AD models [193]. Similarly, targeting NLRP3 inflammasome activation disrupts the downstream NF-κB-mediated cytokine release and reduces pathological aggregates and neuroinflammation, supporting NLRP3 as a viable therapeutic target [194].

Studies have found that optogenetic stimulation of microglia can effectively promote the clearance of Aβ in the brain parenchyma, but it also enhances synaptic elimination. Inhibition of C1q can selectively prevent synaptic loss induced by microglial depolarization without affecting Aβ clearance. Depolarizing microglia and inhibiting complement function to clear antibodies while protecting synapses, can achieve a therapeutic effect [195]. Despite efficacy in preclinical studies, translating microglial-directed immunotherapies to AD patients faces critical dilemmas. First, as mentioned above, the heterogeneity of genes (such as *TREM2* R47H or *APOE4*) determines the direction of disease development to some extent. Thus, a single agonist may not work across *APOE* or *TREM2* genotypes. Furthermore, chronic TREM2 activation can trigger microglial exhaustion or promote a dystrophic phenotype, as seen in the long-term animal study [196]. For STING and NLRP3 inhibitors, systemic blockade of innate immunity may impair the ability to handle opportunistic infections and impaire amyloid clearance in the periphery. Moreover, because immune responses vary across species, positive results in animal experiments cannot be easily included. A critical translational gap is that most studies use young, healthy, genetically homogeneous rodents, whereas human AD patients exhibit chronic, low-grade inflammation with comorbidities (e.g., diabetes, hypertension) that alter microglial setpoints. Therefore, there is still a long way to go from animal models to clinical application. Since monotherapy has certain limitations and faces challenges in clinical translation, multi-target combination therapy may represent a potential treatment strategy in the future.

### α-Syn in PD

**Fig. 4:**
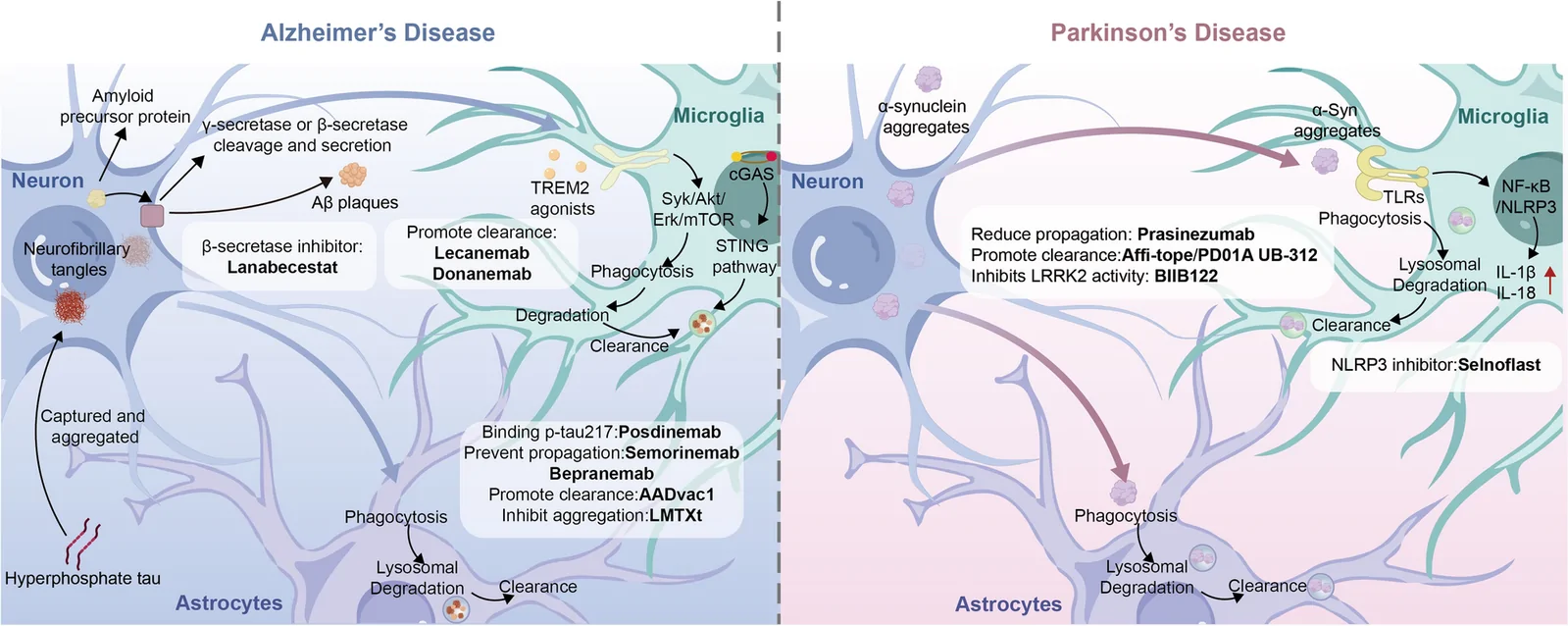
Strategies to clear protein aggregates in AD and PD. Current AD treatment strategies primarily target Aβ and tau. Lanabecestat, a β-secretase inhibitor, primarily reduces Aβ production. Lecanemab and Donanemab primarily promote Aβ clearance. Posdinemab primarily binds to p-tau217 to enhance tau clearance. Semorinemab and bepranemab primarily reduce pathological tau propagation. AADvac1 primarily promotes tau clearance. LMTX inhibits tau aggregate formation. TREM2 agonists act on the TREM2 receptor on microglia, thereby altering the SYK/AKT/ERK/mTOR signaling pathway. This promotes microglia phagocytosis and clearance of Aβ, and reduces excessive tau phosphorylation. Similarly, astrocytes mediate the phagocytosis and clearance of aggregates. Current therapeutic targets for PD primarily focus on the phagocytosis and clearance of α-syn. Among these, prasinezumab primarily reduces the pathological propagation of α-syn, while Affi-tope PD01A and UB-312 mainly promote clearance. BIIB122 is an LRRK2 activity inhibitor that modulates lysosomal function and enhances metabolism. α-Syn aggregates activate the NLRP3 inflammasome. The NLRP3 inhibitor Selnoflast suppresses the TLR/NF-κB/NLRP3 signaling cascade, thereby reducing IL-1β and IL-18 production and mitigating inflammatory responses. Concurrently, astrocytes perform endocytosis and clearance of α-syn

Immunotherapies targeting α-syn (including passive immunity and active vaccination) have been demonstrated in preclinical models to reduce α-syn aggregation pathology. The mechanism likely involves antibody-mediated clearance and inhibition of intercellular spread. Regarding passive immunotherapy, prasinezumab is a humanized IgG1 monoclonal antibody that can selectively bind to the C-terminus of α-syn aggregates, targeting the formation of insoluble fibrils and the formation of Lewy bodies without affecting its monomeric state, and may slow progression by reducing extracellular α-syn and its spread, thereby improving early-stage PD-related pathology. Phase II/III trials have evaluated its safety and α-syn binding in PD [197, 198]. Although prasinezumab showed acceptable safety, the Phase II trial did not meet its primary endpoint, failing to show significant improvement in motor scores compared to placebo. Post-hoc analyses suggested potential benefits in a subgroup with faster disease progression. The heterogeneous patient populations and the lack of reliable α-syn tracers make it difficult to assess target engagementt. These results underscore the need for better patient stratification and biomarkers to identify individuals likely to respond. Active immunotherapy approaches such as AffiTope PD01A and UB-312 primarily function by eliciting an immune response to produce anti-α-syn antibodies within patients, thereby neutralizing extracellular α-syn aggregates, promoting the clearance by microglia, and reducing propagation. In preliminary studies, these therapies reduced α-syn levels in body fluids [199]. However, active vaccination faces unique translational barriers. AffiTope PD01A completed Phase I with acceptable safety but showed highly variable antibody responses, with some patients failing to seroconvert. Moreover, even when antibodies were induced, they did not consistently correlate with clinical stabilization. This likely reflects immune deficiency in the aged PD population, as well as the challenge of breaking immune tolerance to self-proteins (such as α-syn). These limitations suggest that active immunization may require adjuvants or boosting strategies optimized for the immune system in the elderly, and that surrogate endpoints such as CSF α-syn levels may not predict clinical benefit [200]. Targeting the root causes of disease represents the most cutting-edge approach. At doses that are generally safe and well tolerated, BIIB122 potently inhibited peripheral LRRK2 kinase activity and modulated the lysosomal pathway while also providing evidence of CNS distribution and target inhibition [201]. These findings support continued exploration of BIIB122 as an LRRK2 inhibitor for treating PD. BIIB122 is currently under a phase II clinical trial. These findings provide strong support for LRRK2 inhibition as a potential therapeutic strategy to modify the progression of PD [202]. Several translational issues warrant attention. First, *LRRK2* mutations account for approximately 5%–10% of familial PD and less than 2% of sporadic PD, limiting the genetically eligible population unless LRRK2 inhibition also benefits idiopathic patients through a different mechanism. Second, chronic LRRK2 inhibition may cause off-target effects in peripheral organs, as LRRK2 is highly expressed in the lung, kidney, and immune cells. Indeed, some LRRK2 inhibitors have been associated with pulmonary adverse events in animal studies. Third, biomarker evidence of CNS target engagement does not guarantee clinical efficacy, as seen in other kinase inhibitors for NDs. Thus, ongoing Phase II trials must carefully assess the risk–benefit ratio before advancing to Phase III. With regard to the α-syn/TLR/NF-κB/NLRP3 inflammatory pathway, it is well established that α-syn aggregates trigger microglial activation via TLR2, leading to sequential activation of NF-κB and the NLRP3 inflammasome, resulting in the release of pro-inflammatory cytokines such as IL-1β and IL-18 and chronic inflammation. Selnoflast is a potent and selective NLRP3 inflammasome inhibitor that can interrupt this inflammatory cascade, thereby attenuating PD-related pathological changes [161, 203]. However, translation of NLRP3 inhibition in PD patients faces critical hurdles. First, the role of the NLRP3 inflammasome in humans may differ from that in rodent models, as human microglia require a stronger priming signal for full activation. Second, chronic systemic NLRP3 blockade increases the risk of opportunistic infections such as bacterial or fungal infection, as the inflammasome is essential for host defense. Third, Selnoflast and similar inhibitors have shown modest CNS penetration, and the effective brain concentration required to suppress microglial NLRP3 in the context of established α-syn pathology remains uncertain. Furthermore, inflammasome activation may be a downstream event rather than a primary driver. Thus, inhibiting it without removing α-syn aggregates may provide only temporary benefit. These considerations highlight the need for biomarkers of inflammasome activity to select patients and monitor target engagement. Multiple studies targeting α-syn aggregate clearance have failed to achieve satisfactory therapeutic outcomes in phase II trials, underscoring the importance of biomarkers and patient subtyping. PD patients show significant pathological heterogeneity in α-syn, with some exhibiting pathological burden that does not strictly correlate with clinical progression, suggesting the need for stratified treatment strategies [204]. A single approach is unlikely to fully address α-syn pathology, and future therapeutic regimens are shifting toward multimodal combinations integrating immunotherapy, gene regulation, and clearance-enhancing pathways (Fig. 4).

In summary, the failure of clinical translation may be attributed to the following reasons. (1) BBB and target accessibility: the concentration of antibodies in the brain is very low compared to that in serum, and most aggregated α-syn resides intracellularly or within synaptic terminals, limiting antibody access. (2) Animal model limitations: most preclinical studies used α-syn PFF injection models or transgenic mice that rapidly develop widespread pathology within weeks, which does not reflect the slow, progressive, Braak-like spread in humans over decades. These models often overestimate antibody efficacy. (3) Patient heterogeneity and biomarker gap: PD patients exhibit substantial variability in α-syn strain conformation, spreading patterns, and concomitant pathologies. Without a reliable α-syn tracer or validated CSF seeding assay for patient stratification, trials would inevitably include non-responders. (4) Timing of intervention: by the time motor symptoms emerge, 50%–70% of nigral dopaminergic neurons are already lost. Immunotherapies targeting α-syn aggregates cannot reverse neuronal death. Future translation will require development of α-syn tracers and validated CSF assays for patient selection; trials in early-stage or at-risk populations; improved animal models such as human α-syn knock-in mice with aged immune systems; and rational combination strategies that pair immunotherapy with enhancers of lysosomal clearance or neurorestoration. However, combinational therapy itself may introduce complexities in translational medicine, including potential drug interactions, cumulative immunosuppression, and difficulty of attributing efficacy or toxicity to a single component. Future clinical trials will need to focus more on genes or biomarkers.

### ALS and FTD

The main pathological feature of ALS is the loss of motor neurons, leading to muscle weakness, atrophy and eventual paralysis [205]. More than 25 key genes have been found to be involved in ALS pathophysiology, including *SOD1* (superoxide dismutase 1), *C9ORF72*, etc. [206–208]. Mutations in these genes can lead to excitotoxicity, oxidative stress, neuroinflammation, etc. In addition, mutations in the *TDP-43* [209] and *FUS* [210] genes can lead to mislocalization and abnormal aggregation of their proteins [211, 212]. FTD is a neurological disease characterized mainly by neuronal degeneration in the frontal and temporal lobes, with symptoms including behavioral abnormalities, language impairment or executive function decline. FTD also has the main pathological feature of abnormal TDP-43 aggregation. The overlapping clinical and pathological features between FTD and ALS indicating that they belong to the same spectrum of disease [213, 214]. Microglia recognize abnormally aggregated proteins through receptors, engulf them, and degrade them in lysosomes. An overload of abnormal proteins or long-term dysfunction of microglia may impair the functions of lysosomes to degrade proteins, thereby aggravating the pathology [215].

Although there are currently no drugs that can completely cure ALS/FTD, the development of therapeutic drugs for ALS and FTD is developing rapidly. Qalsody (Tofersen) is the first drug approved to treat ALS associated with *SOD1* mutations. Tofersen is an antisense oligonucleotide that induces degradation of *SOD1* mRNA. In a 28-week trial, the intrathecal administration of tofersen reduced plasma concentrations of neurofilament protein (a biomarker of axonal damage) and total SOD1 protein in the CSF of patients with *SOD1* mutation-related ALS, thereby delaying the progression of ALS [216, 217]. However, the success of Tofersen is limited to a small genetic subset. *SOD1* mutations account for only approximately 2% of ALS cases and even fewer FTD cases. Moreover, the requirement for repeated intrathecal injections induces risks of procedure-related complications. Long-term safety data beyond 28 weeks remain limited, and it is unknown whether chronic antisense oligonucleotide administration leads to off-target effects or neurotoxicity. These limitations highlight that Tofersen, while a landmark approval, does not address the vast majority of ALS/FTD patients, particularly those with TDP-43 pathology. Abnormal aggregation of TDP-43 is a common pathological feature of the vast majority of ALS patients and approximately 50% of FTD patients and is an important target for broad-spectrum treatment. CNM-Au8 is a gold nanoparticle catalytic preparation designed to improve cellular energy metabolism and enhance neuronal resistance to oxidative stress and clearance of abnormal proteins. CNM-Au8 is now undergoing phase II/III clinical trials [218, 219]. Sodium phenylbutyrate and taurursodiol (AMX0035) reduce neuronal death, but the effectiveness and the safety of the combination in patients with ALS are unclear. The ALSFRS-R score revealed that compared with the placebo, AMX0035 delayed functional decline during the 24-week treatment period. However, there were no significant differences in secondary outcomes between the groups, leading to the revocation [220]. The failure of AMX0035 in Phase III trial highlights that small, short-term Phase II studies in NDs can yield false-positive results due to the small sample size, regression to the mean, or unblinded assessment biases. Therefore, the results shold be confirmed in large-scale trials before regulatory approval, and surrogate endpoints such as neurofilament levels should be interpreted cautiously. A phase 2b, double-blind, randomized, placebo-controlled trial investigated the efficacy and safety of low-dose IL-2 combined with riluzole. The results showed that this combination tended to reduce mortality, but the results were not statistically significant [221]. Arimoclomol has been shown to improve muscle function and survival in mouse models of various MN diseases. A possible mechanism is that it induces the expression of heat shock proteins Hsp70 and Hsp90, thereby reducing the level of protein aggregates in motor neurons [222]. Studies have shown that peripheral nervous system injury is associated with the inhibition of axonal growth, through activation of the reticulin 4 receptor (RTN4R, or Nogo receptor). NogoA, one of the three isoforms of the Nogo protein, inhibits neurite growth. NogoA appears to be overexpressed in the skeletal muscle of ALS patients. The anti-NogoA antibody Ozanezumab has been shown to delay the progression of ALS [223]. However, the Phase II trial of Ozanezumab failed to elicit a significant difference in the primary endpoint compared to placebo. Possible reasons include that NogoA expression in human ALS muscle may be a compensatory or an epiphenomenon rather than a primary driver of the disease, that the antibody may not adequately penetrate the neuromuscular junction or spinal cord in humans, or that axonal growth promotion alone may be insufficient to overcome the ongoing degeneration in ALS. This failure again illustrates the need for target validation in human tissue by postmortem studies or using imaging biomarkers. Due to the heterogeneity of the disease, the drug development landscape for ALS and FTD is shifting from traditional symptomatic and supportive care toward precisely targeting the core pathological mechanisms of the disease. Future treatments will likely involve combination therapies tailored to the specific genetic background and pathological features of patients, enabling personalized medicine. However, several hurdles must be overcome before personalized combination therapy becomes a reality. First, currently only a minority of patients such as those with *SOD1*, *C9ORF72*, or *FUS* mutations have well-defined molecular targets. For the majority of sporadic patients, the disease drivers remain unknown. Second, regulatory pathways for combination therapies are complex, as each component must demonstrate contribution to efficacy and safety. Third, the high cost of genetic testing, biomarker monitoring, and multiple drugs may limit accessibility. Fourth, the rapid progression of ALS (median survival 3–5 years) leaves a narrow therapeutic window for adaptive trial designs. Despite these challenges, further exploration of the underlying mechanisms and the development of multi-targeted therapies represent a promising approach.

## Conclusions and future directions

Microglia and astrocytes form an integrated clearance network that is essential for maintaining CNS proteostasis. Pathological proteins such as Aβ, tau, α-syn, and TDP-43 are recognized and internalized through phagocytic receptors and subsequently degraded primarily by harmonious mechanisms. Intercellular transfer through EVs and TNTs further links glial cells and neurons, allowing both redistribution and clearance of pathological materials.

Importantly, the protective capacity of this system is highly dependent on glial state. Acute glial activation promotes phagocytosis, degradation, and tissue repair, whereas chronic exposure to pathological proteins progressively impairs receptor-mediated uptake, lysosomal function, and autophagy, furthermore enhancing inflammatory and oxidative responses. This functional transition from homeostatic clearance to maladaptive inflammation may therefore create a vicious cycle in which defective protein clearance promotes further aggregation and neuronal injury, which in turn sustains glial dysfunction.

These observations highlight glial proteostasis as a potential therapeutic target. Rather than simply enhancing phagocytosis, effective interventions may need to simultaneously restore lysosomal and autophagic capacity and maintain a homeostatic glial phenotype. Given the substantial influence of clearing pathways, integrating genetic and cellular characteristics may be critical for developing more precise strategies to restore glial-mediated protein clearance. 

## Data Availability

No datasets were generated or analysed during preparation of this review.

## Abbreviations

Aβ: Amyloid-beta (β-amyloid)
ABCA1: ATP binding cassette subfamily A member 1
AD: Alzheimer’s disease
AKT: Protein kinase B
ALS: Amyotrophic lateral sclerosis
ALSFRS-R: ALS functional rating scale-revised
AnxA2: Annexin A2
APP: Amyloid precursor protein
APOE: Apolipoprotein E
AQP4: Aquaporin-4
ASC: Apoptosis-associated speck-like protein containing a CARD
BACE1: β-site amyloid precursor protein cleaving enzyme 1
BBB: Blood-brain barrier
CARD: Caspase activation and recruitment domain
cGAS: Cyclic GMP-AMP synthase
CMA: Chaperone-mediated autophagy
CNS: Central nervous system
CR1/CR3/CR4: Complement receptor 1/3/4
CSF: Cerebrospinal fluid
DAM: Disease-associated microglia
ER: Endoplasmic reticulum
EVs: Extracellular vesicles
FcγRs: Fc gamma receptors
FTD: Frontotemporal dementia
FTLD: Frontotemporal lobar degeneration
GAS6: Growth arrest-specific 6
GULP1: Engulfment adaptor PTB domain-containing protein 1
GWAS: Genome-wide association studies
HSPGs: Heparan sulfate proteoglycans
IFN-I: Type I interferon
IL: Interleukin
iPSC: Induced pluripotent stem cell
ISF: Interstitial fluid
ITAM: Immunoreceptor tyrosine-based activation motif
ITIM: Immunoreceptor tyrosine-based inhibitory motif
LAG3: Lymphocyte activation gene-3
LAMP1/2A: Lysosomal associated membrane protein 1/2A
LAP: LC3-associated phagocytosis
LC3: Microtubule-associated protein 1A/1B-light chain 3
LCR1: Low-copy repeat 1
LRP1: Low-density lipoprotein receptor-related protein 1
LRRK2: Leucine-rich repeat kinase 2
MEGF10: Multiple epidermal growth factor-like domains protein 10
mTOR: Mechanistic target of rapamycin
mtDNA: Mitochondrial DNA
MS: Multiple sclerosis
NAMPs: Neurodegeneration-associated molecular patterns
NDs: Neurodegenerative diseases
NLRP3: NLR family pyrin domain containing 3
NLRs: Nucleotide-binding oligomerization domain-like receptors
PD: Parkinson’s disease
PFFs: Preformed fibrils
PI3P: Phosphatidylinositol-3-phosphate
PPA: Pathological protein aggregate
PROS1: Protein S
PRR: Pattern recognition receptor
PS: Phosphatidylserine
RAGE: Receptor for advanced glycation end products
ROS: Reactive oxygen species
SIRPα: Signal regulatory protein α
SOD1: Superoxide dismutase 1
STING: Stimulator of interferon genes
SYK: Spleen tyrosine kinase
TAM: Tyro3-Axl-MERTK receptor tyrosine kinase family
TBK1: TANK-binding kinase 1
TDP-43: TAR DNA-binding protein 43
TLR2/4: Toll-like receptor 2/4
TMEM106b: Transmembrane protein 106B
TNF-α: Tumor necrosis factor alpha
TNTs: Tunneling nanotubes
TREM2: Triggering receptor expressed on myeloid cells 2
UPS: Ubiquitin-proteasome system
α-syn: α-synuclein
